# Three New Oleanane-Type Triterpenoidal Glycosides from *Impatiens balsamina* and Their Biological Activity

**DOI:** 10.3390/plants9091083

**Published:** 2020-08-24

**Authors:** Tae Hyun Lee, Won Se Suh, Lalita Subedi, Sun Yeou Kim, Sang Un Choi, Kang Ro Lee, Chung Sub Kim

**Affiliations:** 1 School of Pharmacy, Sungkyunkwan University, Suwon 16419, Korea; thlee16@skku.edu (T.H.L.); wonse528@gmail.com (W.S.S.); krlee@skku.edu (K.R.L.); 2 Gachon Institute of Pharmaceutical Science, Gachon University, Incheon 21936, Korea; subedilali@gmail.com (L.S.); sunnykim@gachon.ac.kr (S.Y.K.); 3 College of Pharmacy, Gachon University, Hambakmoero, Yeonsu-gu, Incheon 21936, Korea; 4 Korea Research Institute of Chemical Technology, Daejeon 34114, Korea; suchoi@krict.re.kr

**Keywords:** *Impatiens balsamina*, balsaminaceae, triterpenoidal glycosides, cytotoxicity, anti-neuroinflammation

## Abstract

Three new oleanane-type triterpenoidal glycosides, imbalosides A–C (**1**–**3**), were isolated from the white flowers of *Impatiens balsamina*. The structures of these phytochemical constituents (**1**–**3**) were elucidated through 1D and 2D Nuclear Magnetic Resonance (NMR) and Mass Spectrometry (MS) data analyses followed by chemical methods. All the characterized compounds (**1**–**3**) were evaluated for their antiproliferative activity against four human tumor cell lines (A549, SK-OV-3, SK-MEL-2, and BT549) and their anti-neuroinflammatory activity on the basis of inhibition levels of nitric oxide (NO) in the lipopolysaccharide (LPS)-stimulated murine microglia BV-2 cell lines.

## 1. Introduction

*Impatiens balsamina* L., known as garden balsam or rose balsam, is an annual plant belonging to the family Balsaminaceae and is widely distributed in Korea, Japan, and mainland China. Diverse parts of *I. balsamina*, including flowers, stems, and leaves, have long been used as traditional medicines to treat various diseases. The flowers of *I. balsamina* have been used as remedies for lumbago, burns, and scalds [1], whereas its aerial parts have been used to treat articular rheumatism, abscesses, and tumors [2]. In the previous research on this plant, 1,4-naphthoquinone derivatives showed a variety of pharmacological effects such as antitumor, anti-inflammatory, and hepatoprotective activities [3,4,5].

**Figure 1:**
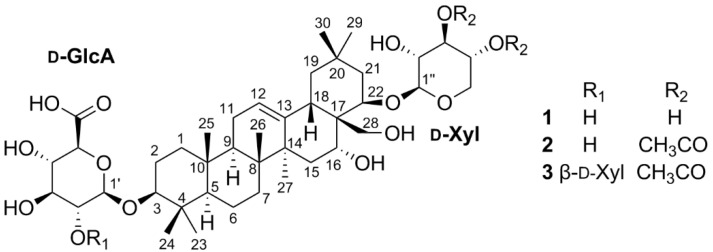
Chemical structures of compounds **1**–**3**.

As part of the continuing studies to identify the bioactive constituents from Korean medicinal plants [6,7,8,9,10,11], we previously conducted a phytochemical investigation on the MeOH extract of the white flowers of *I. balsamina*, which led to the isolation and characterization of phenolic compounds including mono- and biflavonoids with cytotoxic, anti-inflammatory, and neuroprotective activities [12,13]. In order to discover bioactive molecules in other structural classes from this plant, we further investigated its EtOAc-soluble layer and identified three new oleanane-type triterpenoidal glycosides (**1**–**3**) (Figure 1). The chemical structures of the new compounds (**1**–**3**) were established on the basis of spectroscopic (1D and 2D NMR) and spectrometric [High Resolution Fast Atom Bombardment MS (HRFABMS)] analyses as well as chemical methods. The isolates (**1**–**3**) were tested for their cytotoxicity against four human tumor cell lines and anti-neuroinflammatory activity using lipopolysaccharide (LPS)-stimulated murine microglia BV-2 cell lines.

## 2. Results and Discussion

### 2.1. Structure Elucidation

**Figure 2:**
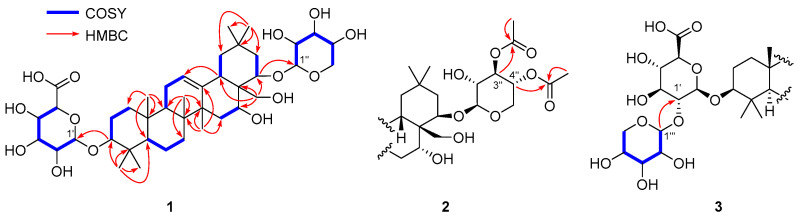
Key COSY (blue bold) and HMBC (red arrows) correlations of **1**–**3**.

Imbaloside A (**1**) was isolated as a colorless gum. The molecular formula of **1** was determined as C_41_H_66_O_14_ based on the [M – H]^–^ ion peak at *m/z* 781.4368 (calcd for C_41_H_65_O_14_^–^, 781.4380, error = 1.5 ppm) from the HRFABMS analysis. The ^1^H NMR spectrum of **1** showed a broad singlet at δ_H_ 5.30 for an olefinic proton, overlapped signals from δ_H_ 3.20 to 4.38 for oxygenated methine/methylene protons, seven singlets at δ_H_ 1.40, 1.09, 1.05, 1.00, 0.97, 0.93, and 0.88 for methyl protons, and the others in the region from δ_H_ 2.28 to 0.82. Among a total of 41 carbons present in this molecule, 40 resonances were observed in the ^13^C NMR spectrum of **1**, including 30 peaks for typical oleanane-type triterpenoids [14,15,16,17,18] with two olefinic carbons at δ_C_ 144.6 and 124.2 and four oxygenated carbons at δ_C_ 91.1, 78.8, 70.4, and 65.8, and ten peaks for β-glucuronic acid (δ_C_ 106.9, 75.6, 78.1, 73.8, and 76.8) and β-xylopyranose (δ_C_ 102.8, 74.5, 77.0, 71.2 and 66.5). These ^1^H and ^13^C NMR data implied that **1** is an oleanane-type triterpenoidal glycoside with two sugar moieties, and its core structure including the location of a double bond and four oxygenated carbons was established through analysis of the Distortionless Enhancement by Polarization Transfer (DEPT), correlation spectroscopy (COSY), Heteronuclear Single Quantum Correlation (HSQC), and Heteronuclear Multiple Bond Correlation (HMBC) spectra (Figure 2, Supplementary Materials).

**Figure 3:**
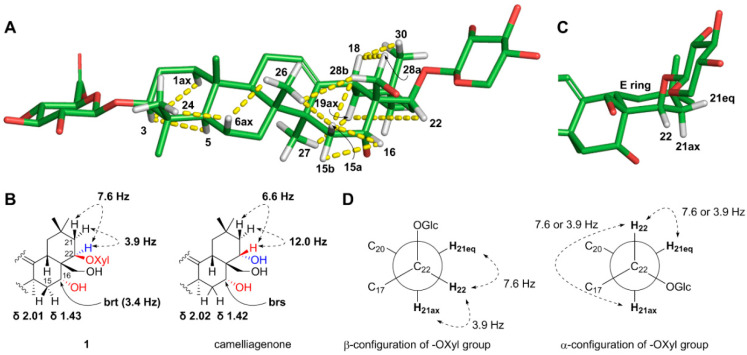
Stereochemical analysis of **1**. (**A**) Key NOESY (yellow dashed) correlations of **1**. The geometry of the 3D structure was minimized at the MMFF force field. Some protons were removed for a clearer presentation. (**B**) Comparison of selected chemical shifts and coupling constants of **1** and camelliagenone, which possesses the same α-OH at C-16 and the opposite α-OH at C-22 to those of **1**. (**C**) Zoomed-in view around the E ring in **1**. (**D**) Newman projections from C-22 to C-21 for the two possible 22-epimers and the observed coupling constants between H-22 and H-21eq,ax.

The β-configurations of the two anomeric carbons on the glucuronic acid and xylopyranose were assigned by the relatively large ^3^*J* coupling constants (7.1 Hz) between H-1 and H-2 of both sugars. These two sugar units were confirmed to be connected at C-3 (glucuronic acid) and C-22 (xylopyranose) by observing HMBC cross-peaks of H-3/C-1′ and H-22/C-1′′ (Figure 2). The relative configuration at C-3 was confirmed by the strong nuclear Overhauser effect (NOE) correlation of H-3 with H-5 along with the mild correlation of H-3 with H-1ax (Figure 3A). The α-orientation of the hydroxyl group at C-16 was verified by the strong NOE spectroscopy (NOESY) cross-peaks of H-16 with both H-15a and H-15b (Figure 3) and the relatively small coupling constant, 3.4 Hz, between H-16 and both H-15a and H-15b, indicating that H-16 should be in equatorial position rather than axial position. This initial assignment of α-OH at C-16 was supported by observing a similar NMR chemical shift pattern around C-16 of camelliagenone (e.g., δ_H-15_ 2.02 and 1.42 for camelliagenone and δ_H-15_ 2.01 and 1.43 for **1**) and the coupling constant (broad singlet at C-16 for camelliagenone and broad triplet with the small coupling constant 3.4 Hz for **1**), which possess the same α-orientation of the hydroxyl group at C-16 [19]. The β-configuration of the alkoxy group at C-22 was assigned by NOESY cross-peak of H-22 with H-19ax (Figure 3A) and coupling constant analysis (Figure 3B,C); the Newman projections of two possible 22-epimers suggested that both H-22/H-21eq and H-22/H-21ax are *gauche* in the β-OXyl epimer, whereas H-22/H-21ax is *anti* and H-22/21eq is *gauche* in the α-OXyl epimer (Figure 3C). Generally, the coupling constant of two protons in *anti* (or axial–axial) orientation in cyclohexane is over 9–10 Hz, which excluded the possibility of an α-OXyl epimer for **1** since the observed coupling constants between H-22 and H-21ax,eq were both smaller than 8 Hz (7.6 and 3.9 Hz, respectively). In fact, camelliagenone has an α-OH at C-22 and the coupling constant between H-22 and H-21α was 12.0 Hz [19]. The hydroxymethyl group at C-28 was confirmed as β-form by the NOE correlations of H-28a with H-18 and H-28b with H-15 and H-26 (Figure 3A). The absolute configurations of the glucuronic acid and xylopyranose were assigned as D-form by comparing retention times of their chiral derivatives with those of authentic samples [20]. Thus, the structure of **1** was defined as 3-*O*-β-D-glucuronyl-3β,16α,22β,28-tetrahydroxyolean-12-ene-22-*O*-β-D-xylopyranoside.

Imbaloside B (**2**) was obtained as a colorless gum, and its molecular formula was determined as C_45_H_70_O_16_ from the deprotonated HRFABMS ion peak at *m/z* 865.4580 (calcd for C_45_H_69_O_16_^–^, 865.4591, error = 1.3 ppm). The ^1^H and ^13^C NMR data were quite similar to those of compound **1** (Table 1), suggesting that compound **2** possesses the same core structure as compound **1**, but apparent differences existed at the β-D-xylopyranose moiety in terms of the presence of two acetoxy groups (δ_H_ 2.08 and 2.02; δ_C_ 172.2, 171.8, 21.0 and 20.7) (Table 1). The connectivity of the two acetoxy groups were verified on the basis of the HMBC cross-peaks from H-3′′ (δ_H_ 5.09) to OAc-3′′ (δ_C_ 172.2) and H-4’’ (δ_H_ 4.87) to OAc-4’’ (δ_C_ 171.8), respectively (Figure 2). According to the similar ^1^H and ^13^C NMR data and NOESY correlations to those of compound **1**, the relative configuration of compound **2** was confirmed to be identical with that of **1**. Thus, the chemical structure of **2** was assigned as 3-*O*-β-D-glucuronyl-3β,16α,22β,28-tetrahydroxyolean-12-ene-22-*O*-(3,4-*O*-diacetyl)-β-D-xylopyranoside.

Imbaloside C (**3**) was isolated as a colorless gum, with the molecular formula C_50_H_78_O_20_, by the deprotonated HRFABMS ion peak at *m/z* 997.5002 (calcd for C_50_H_77_O_20_^–^, 997.5014, error = 1.2 ppm). Inspection of the ^1^H and ^13^C NMR data of **3** proposed that these data closely resembled those of **2**, with a noticeable difference of the presence of one set of resonances attributable to a xylopyranose unit [δ_H_ 4.55, 3.84, 3.49, 3.35, 3.26, and 3.17; δ_C_ 106.5, 77.9, 76.5, 71.4, and 67.3] with a β-configuration suggested by the large coupling constant (7.4 Hz) between H-1′′′ and H-2′′′ (Table 1). The location of this additional xylopyranose unit was determined by the HMBC cross-peak of H-1′′′ (δ_H_ 4.55) to C-2′ (δ_C_ 83.4) (Figure 2). Moreover, detailed inspection of the NOESY correlations verified that the relative configuration of **3** was all the same as those of **1** and **2**. Thus, the structure of **3** was elucidated as 3-*O*-[β-D-xylopyranosyl-(1→2)]-β-D-glucuronyl-3β,16α,22β,28-tetrahydroxyolean-12-ene-22-*O*-(3,4-*O*-diacetyl)-β-D-xylopyranoside.

### 2.2. Cytotoxicity Assessment

The cytotoxicity was assessed based on the inhibitory effects of the compounds (**1**–**3**) on the growth of the four human tumor cell lines A549, SK-OV-3, SK-MEL-2, and BT549 using a sulforhodamine B (SRB) assay. As shown in Table 2, imbaloside B (**2**) displayed weak cytotoxicity against the BT549 cell line with an IC_50_ value of 26.4 μM, whereas it was inactive against the other cell lines (IC_50_ > 30 μM). Imbaloside C (**3**) showed mild cytotoxic activities against the A549 and BT549 cell lines with IC_50_ values of 29.8 and 29.2 μM, respectively. Cisplatin was used as a positive control with IC_50_ values of 0.9–2.0 μM against the four tumor cell lines (Table 2).

### 2.3. Anti-Neuroinflammatory Activity

The potential anti-neuroinflammatory activity of the new compounds (**1**–**3**) was also evaluated by measuring the nitric oxide (NO) production levels in the LPS-stimulated murine microglia BV-2 cell line. The tested compounds (**1**–**3**) exerted moderate inhibition levels of NO production with IC_50_ values ranging from 33.8 to 41.0 μM without significant cell toxicity. L-NMMA (IC_50_ 21.3 μM) was used as a positive control (Table 3).

Many oleanane-type triterpenoids and their glycosides have shown potent cytotoxicity and anti-neuroinflammatory activity that are consistent with our current study [16,21,22,23,24]. This implied that the well-known oleanane-type triterpenoids are still good sources for future drug candidates to treat cancer or inflammation-related diseases.

## 3. Materials and Methods

### 3.1. General Experimental Procedures

Optical rotation data were recorded using a JASCO P-1020 polarimeter (JASCO, Easton, MD, USA). The NMR studies were accomplished employing a Bruker AVANCE III 700 NMR spectrometer (Bruker, Karlsruhe, Germany) and resultant spectra were processed using MestReNova (Mnova) (version 14.1.2-25024) with default weighting functions. HRFABMS data were acquired on a Waters SYNAPT G2 (Milford, MA, USA). The HPLC-DAD-MS data were measured using an Agilent 1260 Infinity HPLC system (Agilent, Santa Clara, CA, USA) with a Kinetex C_18_ 5 µm column (250 mm length × 4.6 mm i.d.; Phenomenex, Torrance, CA, USA). Purification was achieved using a semi-preparative HPLC system equipped with a Gilson 306 pump (Middleton, WI, USA), a Shodex refractive index detector (New York, NY, USA), and a Luna C_18_ 10 µm column (250 mm length × 10 mm i.d.; Phenomenex, Torrance, CA, USA). Low-pressure liquid chromatography (LPLC) was performed with a LiChroprep Lobar-A Si 60 column (Merck, Darmstadt, Germany) and an FMI QSY-0 pump (Teledyne Isco, Lincoln, NE, USA). Open columns packed with silica gel 60 (70–230 and 230–400 mesh; Merck) or RP-C18 silica gel (230–400 mesh; Merck, Darmstadt, Germany) were implemented for crude fractionation and separation. Precoated silica gel F254 plates and RP-18 F254s plates (Merck) were utilized for thin-layer chromatography (TLC).

### 3.2. Plant Material

The air-dried white flowers of *I. balsamina* were collected in Asan, Korea, in August 2014, and the plant was identified by one of the authors (K.R.L.). A voucher specimen (SKKU-NPL 1406) was deposited in the herbarium of the School of Pharmacy, Sungkyunkwan University, Suwon, Korea.

### 3.3. Extraction and Isolation

The white flowers of *I. balsamina* (3.0 kg) were extracted with 80% aqueous MeOH under reflux and filtered. The filtrate was concentrated under a reduced pressure to obtain a MeOH extract (730 g). The crude extract was suspended in distilled H_2_O and successively partitioned with *n*-hexane, CHCl_3_, EtOAc, and *n*-butanol, yielding 62, 55, 50, and 86 g of the respective solvent residues. The EtOAc-soluble fraction (20 g) was separated over a silica gel column (CHCl_3_-MeOH-H_2_O, 4:1:0.1) to yield eight fractions (A–H). Fraction E (1.5 g) was chromatographed on an RP-C_18_ silica gel column (55% aqueous MeOH) to yield nine subfractions (E1–E9). Compounds **2** (5 mg) and **3** (3 mg) were obtained from subfraction E8 (230 mg) using a Lobar-A Si 60 column (CHCl_3_-MeOH-H_2_O, 4:1:0.1) followed by semi-preparative HPLC (30% aqueous MeCN). Fraction F (1.0 g) was applied to an RP-C_18_ silica column (55% aqueous MeOH) and further purified by semi-preparative HPLC (25% aqueous MeCN) to afford compound **1** (10 mg).

#### 3.3.1. Imbaloside A (**1**)

Colorless gum; ${\left[\alpha \right]}_{D}^{25}$ +11 (c 0.1, MeOH); ^1^H (700 MHz) and ^13^C NMR (175 MHz) data, see Table 1; HRFABMS (negative-ion mode) *m/z* 781.4368 [M – H]^–^ (calcd for C_41_H_65_O_14_^–^, 781.4380, error = 1.5 ppm).

#### 3.3.2. Imbaloside B (**2**)

Colorless gum; ${\left[\alpha \right]}_{D}^{25}$ +15 (c 0.1, MeOH); ^1^H (700 MHz) and ^13^C NMR (175 MHz) data, see Table 1; HRFABMS (negative-ion mode) *m/z* 865.4580 [M – H]^–^ (calcd for C_45_H_69_O_16_^–^, 865.4591, error = 1.3 ppm).

#### 3.3.3. Imbaloside C (**3**)

Colorless gum; ${\left[\alpha \right]}_{D}^{25}$ +30 (c 0.2, MeOH); ^1^H (700 MHz) and ^13^C NMR (175 MHz) data, see Table 1; HRFABMS (negative-ion mode) *m/z* 997.5002 [M – H]^–^ (calcd for C_50_H_77_O_20_^–^, 997.5014, error = 1.2 ppm).

### 3.4. Acid Hydrolysis of 1–3 and Sugar Analysis

Compounds **1**–**3** (1 mg) were individually hydrolyzed with 1 N HCl (1 mL) under reflux for 2 h. CHCl_3_ was used to extract organic layers from each reaction mixture. The monosaccharides acquired from H_2_O-soluble phases were added to pyridine (0.5 mL) containing L-cysteine methyl ester hydrochloride (0.5 mg) and the respective reaction mixtures were stirred at 60 °C for 1 h. Then, *o*-tolyl isothiocyanate (0.1 mL) was added and stirred at 60 °C for another 1 h. Each reaction mixture was analyzed without purification by LC-MS analysis (0.7 mL/min; 25% aqueous CH_3_CN with 0.1% formic acid for 30 min). The authentic samples of D-xylopyranose, L-xylopyranose, and D-glucuronic acid were derivatized and analyzed by the same method as described above. Since standard L-glucuronic acid was not commercially available, we derivatized D-glucuronic acid with enantiomeric D-cysteine methyl ester hydrochloride to deduce the retention time of the L-glucuronic acid derivative. The hydrolysate derivatives of **1**–**3** were detected at 23.3 min for D-xylopyranose and 20.4 min for D-glucuronic acid in the LC-MS analysis, which corresponded with those of D-forms of authentic sugars (23.3 min for D-xylopyranose, 21.4 min for L-xylopyranose, 20.4 min for D-glucuronic acid, and 19.6 min for L-glucuronic acid).

### 3.5. Cytotoxicity Assessment

The cytotoxicity of the purified metabolites was tested against the A549 (non-small cell lung adenocarcinoma), SK-OV-3 (ovary malignant ascites), SK-MEL-2 (skin melanoma), and BT549 (invasive ductal carcinoma), utilizing the sulforhodamine B (SRB) colorimetric method [25]. Cisplatin (≥98%; Sigma-Aldrich, St. Louis, MO, USA) served as a positive control.

### 3.6. Assessment of NO Generation and Cell Viability

The BV-2 cells, developed by Dr. V. Bocchini at the University of Perugia (Perugia, Italy), were used for this study [26,27]. The cells were seeded in a 96-well plate (4 × 104 cells/well) and incubated in the presence or absence of various doses of the tested compounds. Lipopolysaccharide (LPS) (100 ng/mL) was added to BV-2 cells and grown for 1 d. The produced levels of nitrite (NO_2_), a soluble oxidized product of NO, were evaluated with 0.1% N-1-napthylethylenediamine dihydrochloride and 1% sulfanilamide in 5% phosphoric acid, also known as the Griess reagent. The supernatant (50 μL) was mixed with the Griess reagent (50 μL). After 10 min, the absorbance was gauged at 570 nm. For a positive control, the reported nitric oxide synthase (NOS) inhibitor L-NMMA was employed. Graded sodium nitrite solution was utilized to determine nitrite concentrations. An MTT assay was used for the cell viability assay.

## 4. Conclusions

In this study, we described the characterization of three new oleanane-type triterpenoidal glycosides (**1**–**3**) using spectroscopic and spectrometric data analyses and chemical methods. It is the first to report oleanane-type triterpenoids from *I. balsamina*, whereas two other types of triterpenoids, baccharane [28,29,30,31] and ursane [32], were known from this plant. Although further studies are needed, the cytotoxicity and inhibitory potency on NO production of the isolated compounds (**1**–**3**) indicated that these compounds would be potential drug candidates.

## Figures and Tables

**Table 1:** ^1^H [ppm, mult., (*J* in Hz)] and ^13^C NMR data of compounds **1**–**3** in methanol-*d*_4_.

Position	1		2		3	
	*δ*_C_	*δ*_H_	*δ*_C_	*δ*_H_	*δ*_C_	*δ*_H_
1ax	40.2	1.65, overlap	40.2	1.65, overlap	40.3	1.64, overlap
1eq		1.01, overlap		1.03, overlap		1.02, overlap
2ax	27.1	1.72, overlap	27.1	1.73, overlap	27.2	1.73, overlap
2eq		1.97, overlap		2.00, overlap		2.02, overlap
3	91.1	3.21, overlap	91.1	3.22, overlap	91.0	3.21, overlap
4	40.4		40.4		40.5	
5	57.3	0.82, d (11.6)	57.3	0.82, d (11.6)	57.3	0.80, d (11.5)
6ax	19.5	1.44, overlap	19.5	1.46, overlap	19.5	1.44, overlap
6eq		1.61, overlap		1.61, overlap		1.61, overlap
7a	34.4	1.64, overlap	34.4	1.63, overlap	34.4	1.63, overlap
7b		1.43, overlap		1.42, overlap		1.43, overlap
8	41.0		41.0		41.0	
9	48.3	1.66, overlap	48.3	1.67, overlap	48.3	1.66, overlap
10	38.0		38.0		37.9	
11	24.7	1.91, overlap	24.7	1.92, overlap	24.7	1.92, overlap
12	124.2	5.30, brs	124.2	5.30, brt (3.7)	124.2	5.30, brt (3.7)
13	144.6		144.6		144.6	
14	42.7		42.7		42.7	
15ax	34.5	2.01, brd (13.5)	34.6	2.02, overlap	34.6	2.04, overlap
15eq		1.43, overlap		1.43, overlap		1.44, overlap
16	70.4	4.25, brt (3.4)	70.5	4.24, brt (3.5)	70.5	4.23, brt (3.4)
17	45.6		45.6		45.6	
18	42.8	2.06, brd (13.0)	42.8	2.10, d (13.2)	42.8	2.10, d (13.1)
19ax	48.2	2.28, t (13.4)	48.2	2.29, t (13.5)	48.2	2.29, t (13.4)
19eq		1.00, overlap		1.00, overlap		1.01, overlap
20	31.1		31.1		31.1	
21ax	40.2	1.74, dd (13.6, 3.4)	40.4	1.78, dd (13.7, 3.9)	40.4	1.78, dd (13.7, 3.9)
21eq		1.65, overlap		1.69, overlap		1.69, overlap
22	78.8	4.28, dd (7.6, 3.9)	78.8	4.28, dd (7.0, 3.9)	79.8	4.28, dd (7.0, 3.9)
23	28.7	1.09, s	28.7	1.09, s	28.4	1.09, s
24	17.2	0.88, s	17.2	0.89, s	16.8	0.88, s
25	16.4	1.00, s	16.4	1.01, s	16.4	1.01, s
26	17.7	0.97, s	17.7	0.98, s	17.7	0.98, s
27	27.3	1.40, s	27.4	1.41, s	27.4	1.40, s
28a	65.8	3.64, d (12.0)	65.8	3.65, d (12.0)	65.9	3.65, d (12.1)
28b		3.27, overlap		3.29, d (12.0)		3.29, d (12.0)
29	32.6	0.93, s	32.8	0.93, s	32.8	0.93, s
30	29.1	1.05, s	28.9	1.05, s	28.9	1.05, s
1′	106.9	4.38, d (7.1)	106.9	4.38, d (7.8)	105.5	4.47, d (7.6)
2′	75.6	3.28, overlap	75.7	3.27, overlap	83.4	3.51, overlap
3′	78.1	3.41, overlap	78.2	3.42, overlap	78.3	3.61, overlap
4′	73.8	3.50, overlap	73.9	3.49, overlap	73.8	3.51, overlap
5′	76.8	3.68, overlap	76.9	3.63, overlap	77.1	3.61, overlap
6′	n/d		n/d		n/d	
1′′	102.8	4.38, d (7.1)	103.1	4.49, d (7.5)	103.1	4.49, d (7.6)
2′′	74.5	3.23, overlap	72.8	3.44, dd (9.1, 7.5)	72.8	3.44, overlap
3′′	77.0	3.41, overlap	75.9	5.09, t (9.1)	75.9	5.09, t (9.1)
4′′	71.2	3.52, overlap	70.8	4.87, td (9.6, 5.5)	70.8	4.87, td (9.6, 5.4)
5′′ax	66.5	3.29, overlap	63.6	3.46, dd (11.6, 10.0)	63.6	3.46, overlap
5′′eq		3.95, dd (11.6, 4.9)		4.06, dd (11.6, 5.5)		4.06, dd (11.6, 5.4)
OAc-3′′			172.2		172.2	
			21.0	2.08, s	21.0	2.08, s
OAc -4′′			171.8		171.8	
			20.7	2.02, s	20.7	2.02, s
1′′′					106.5	4.55, d (7.4)
2′′′					76.5	3.26, dd (8.9, 7.4)
3′′′					77.9	3.35, overlap
4′′′					71.4	3.49, overlap
5′′′ax					67.3	3.17, overlap
5′′′eq						3.84, dd (11.5, 5.3)

**Table 2:** Cytotoxicity of compounds **1**–**3** against four cultured human cancer cell lines in the sulforhodamine B (SRB) bioassay.

Compound	IC_50_ (μM) ^1^			
	A549	SK-OV-3	SK-MEL-2	BT549
**1**	>30	>30	>30	>30
**2**	>30	>30	>30	26.4
**3**	29.8	>30	>30	29.2
Cisplatin ^2^	0.9	2.0	1.1	1.2

^1^ 50% inhibitory concentration; the concentration of a compound that caused a 50% inhibition in cell growth. ^2^ Positive control substance.

**Table 3:** Inhibitory effect of compounds **1**–**3** on nitric oxide (NO) production in LPS-activated BV-2 cells.

Compound	IC_50_ (μM) ^1^	Cell viability (%) ^2^
**1**	41.0	109.2 ± 6.0
**2**	33.8	97.5 ± 2.1
**3**	34.8	83.7 ± 3.8
L-NMMA ^3^	21.3	120.1 ± 11.7

^1^ The IC_50_ value of each compound is defined as the concentration (μM) that caused 50% inhibition of NO production in LPS-activated BV-2 cells. ^2^ The cell viability following treatment with 20 μM of each compound was determined using an MTT assay and is expressed as a percentage (%). Data are expressed as the mean ± SD of three independent experiments. ^3^ Positive control substance.

## Supplementary Materials

The following are available online at https://www.mdpi.com/2223-7747/9/9/1083/s1, NMR and HRMS data of **1**–**3**.
