# Supplementary material for: Three New Oleanane-Type Triterpenoidal Glycosides from Impatiens balsamina and Their Biological Activity
Source: Plants (Basel). 2020 Aug 24;9(9):1083. doi: 10.3390/plants9091083 (PMC7570163; doi:10.3390/plants9091083)

Supporting Information for

Three new oleanane-type triterpenoidal glycosides from *Impatiens balsamina* and their biological activity

Tae Hyun Lee [1], Won Se Suh [1], Lalita Subedi [2,3], Sun Yeou Kim [2,3], Sang Un Choi [4], Kang Ro Lee [1] and Chung Sub Kim [1,*]

[1] School of Pharmacy, Sungkyunkwan University, Suwon 16419, Republic of Korea; thlee16@skku.edu (T.H.L.); wonse528@gmail.com (W.S.S.); krlee@skku.edu (K.R.L.)

[2] Gachon Institute of Pharmaceutical Science, Gachon University, Incheon 21936, Republic of Korea; subedilali@gmail.com (L.S.); sunnykim@gachon.ac.kr (S.Y.K.)

[3] College of Pharmacy, Gachon University, #191, Hambakmoero, Yeonsu-gu, Incheon 21936, Republic of Korea; subedilali@gmail.com (L.S.); sunnykim@gachon.ac.kr (S.Y.K.)

[4] Korea Research Institute of Chemical Technology, Daejeon 34114, Republic of Korea; suchoi@krict.re.kr (S.U.C.)

[*] *Correspondence: chungsub.kim@skku.edu; Tel.: +82-31-290-7727*

Contents

Figure S1. The HRFABMS spectrum of **1** 3
Figure S2. The ^{1}H NMR spectrum of **1** in methanol-*d*4 4
Figure S3. The ^{13}C NMR spectrum of **1** in methanol-*d*4 5
Figure S4. The COSY spectrum of **1** in methanol-*d*4 6
Figure S5. The HSQC spectrum of **1** in methanol-*d*4 7
Figure S6. The HMBC spectrum of **1** in methanol-*d*4 8
Figure S7. The NOESY spectrum of **1** in methanol-*d*4 9
Figure S8. The DEPT90 (top) and 135 (bottom) spectra of **1** in methanol-*d*4 10
Figure S9. The HRFABMS spectrum of **2** 11
Figure S10. The ^{1}H NMR spectrum of **2** in methanol-*d*4 12
Figure S11. The ^{13}C NMR spectrum of **2** in methanol-*d*4 13
Figure S12. The COSY spectrum of **2** in methanol-*d*4 14
Figure S13. The HSQC spectrum of **2** in methanol-*d*4 15
Figure S14. The HMBC spectrum of **2** in methanol-*d*4 16
Figure S15. The NOESY spectrum of **2** in methanol-*d*4 17
Figure S16. The HRFABMS spectrum of **3** 18
Figure S17. The ^{1}H NMR spectrum of **3** in methanol-*d*4 19
Figure S18. The ^{13}C NMR spectrum of **3** in methanol-*d*4 20
Figure S19. The COSY spectrum of **3** in methanol-*d*4 21
Figure S20. The HSQC spectrum of **3** in methanol-*d*4 22
Figure S21. The HMBC spectrum of **3** in methanol-*d*4 23
Figure S22. The NOESY spectrum of **3** in methanol-*d*4 24

Figure S1. The HRFABMS spectrum of **1**

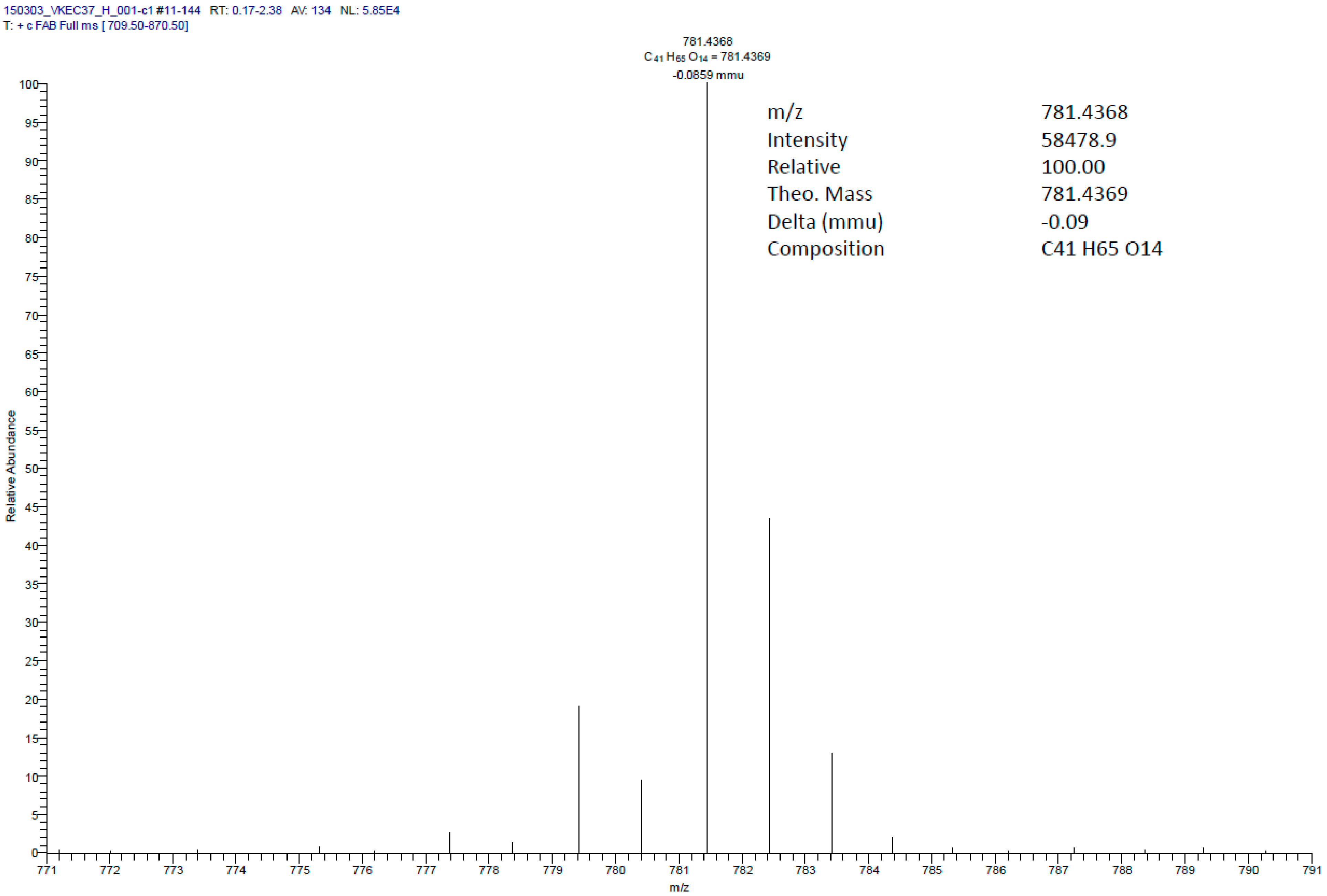

Figure S2. The ^{1}H NMR spectrum of **1** in methanol-d_4

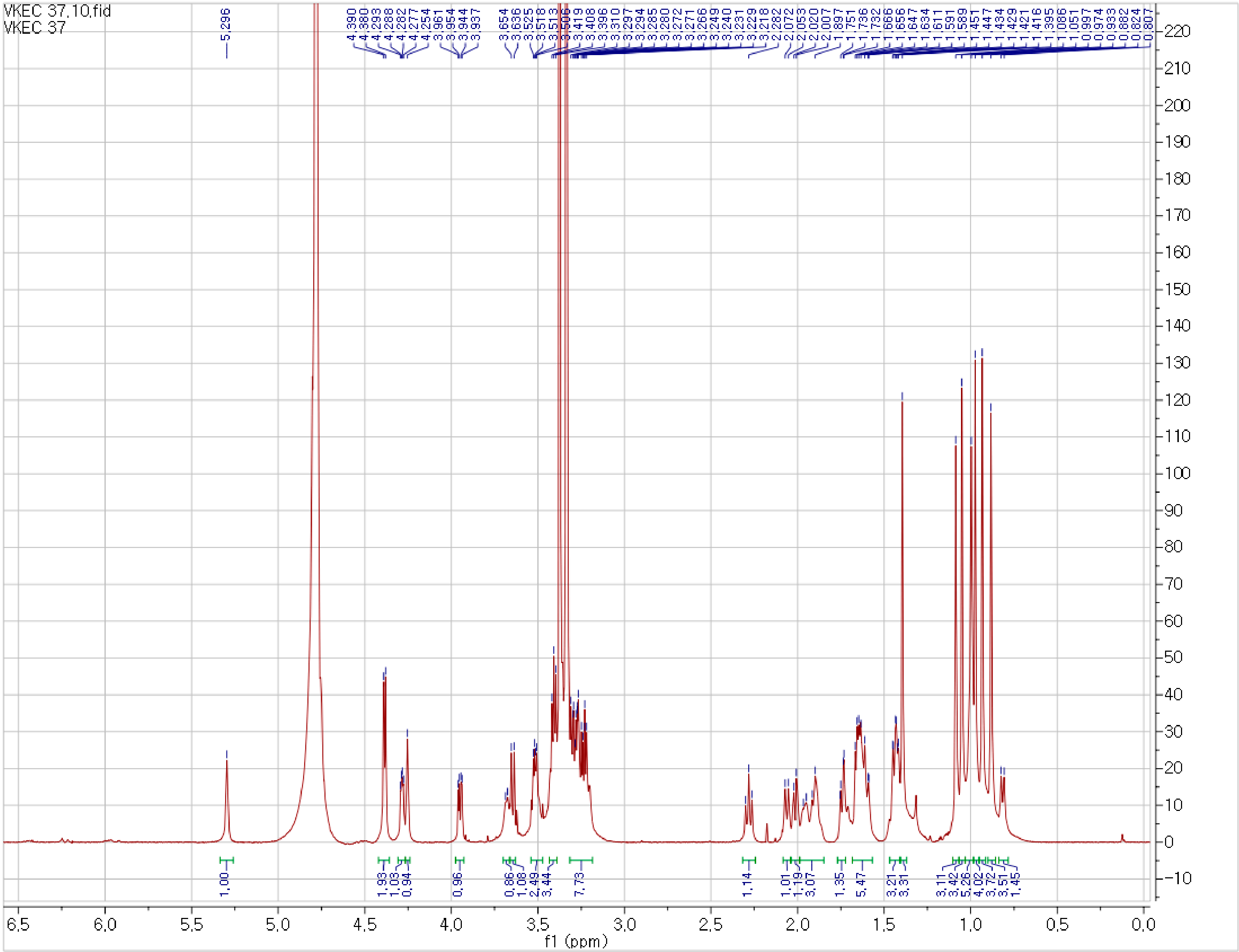

Figure S3. The ^{13}C NMR spectrum of **1** in methanol-*d*4

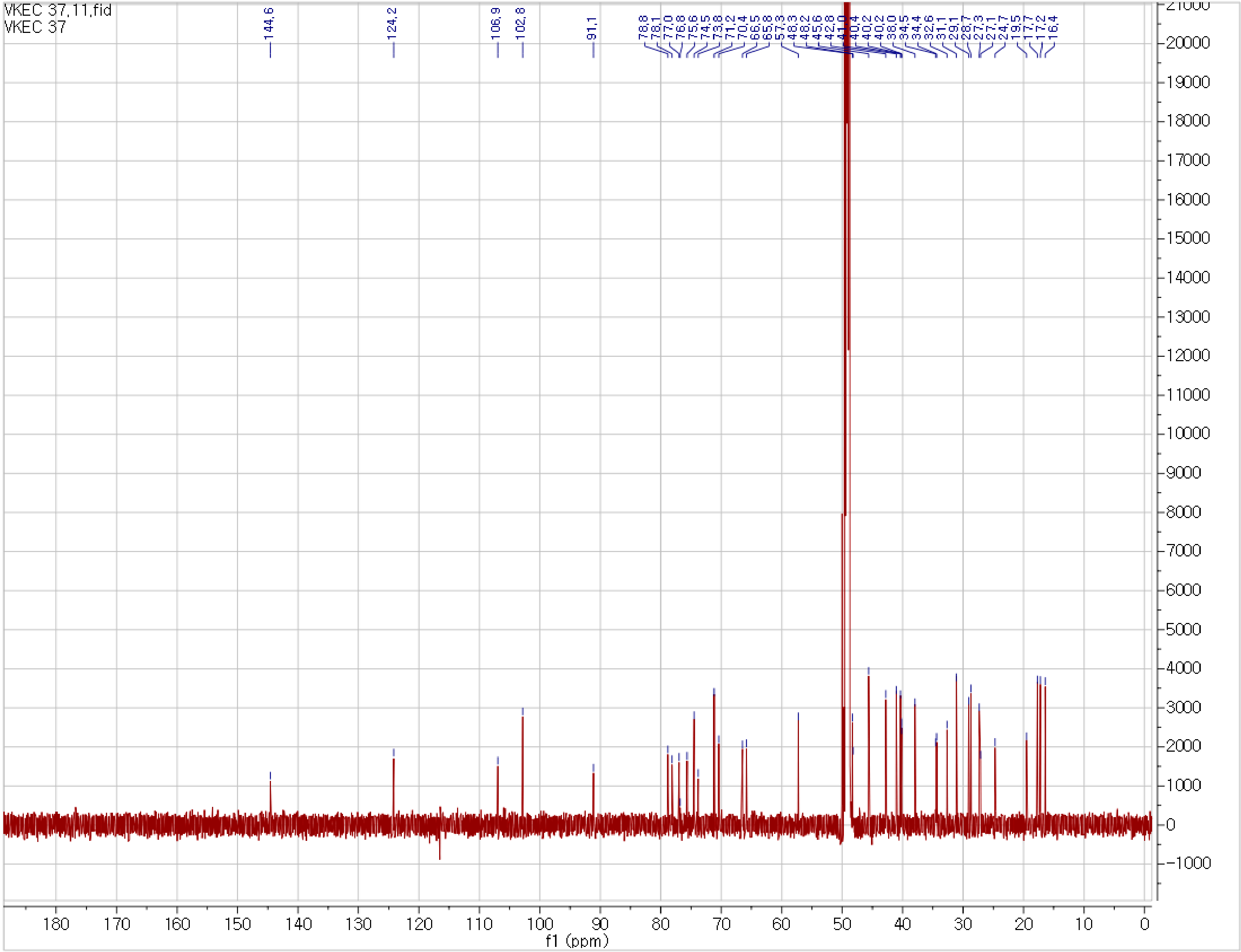

Figure S4. The COSY spectrum of **1** in methanol-d_4

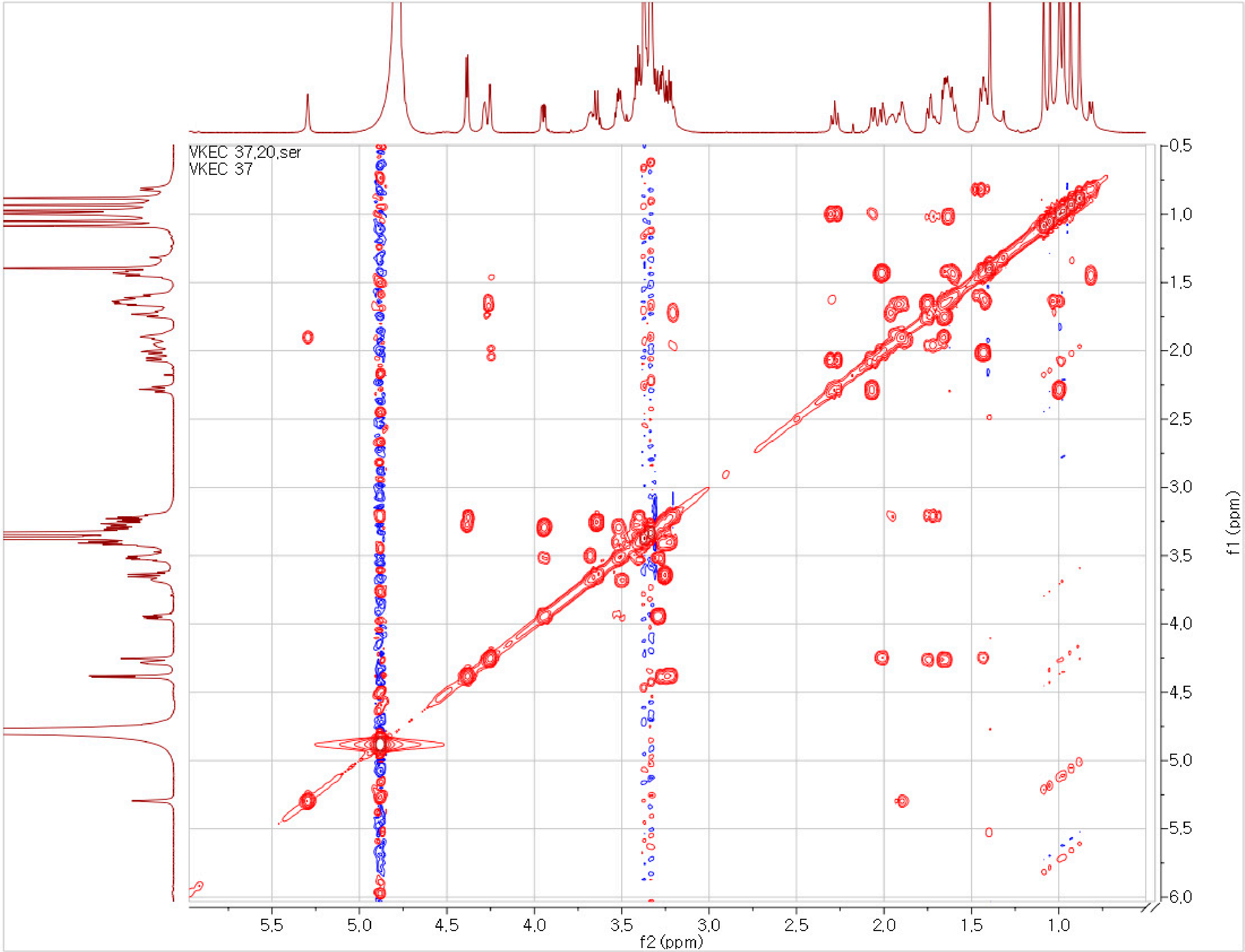

Figure S5. The HSQC spectrum of **1** in methanol-*d*4

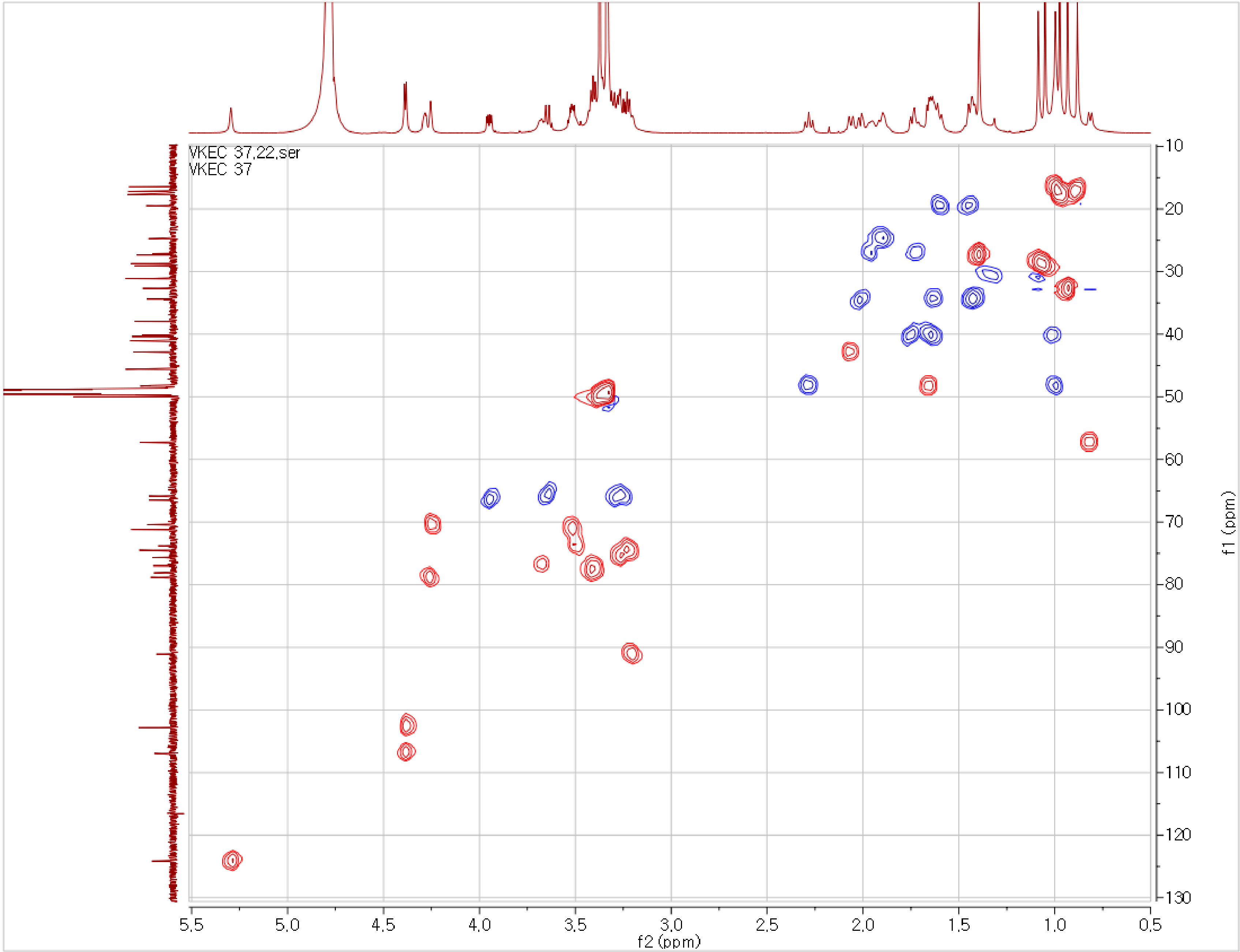

Figure S6. The HMBC spectrum of **1** in methanol-*d*4

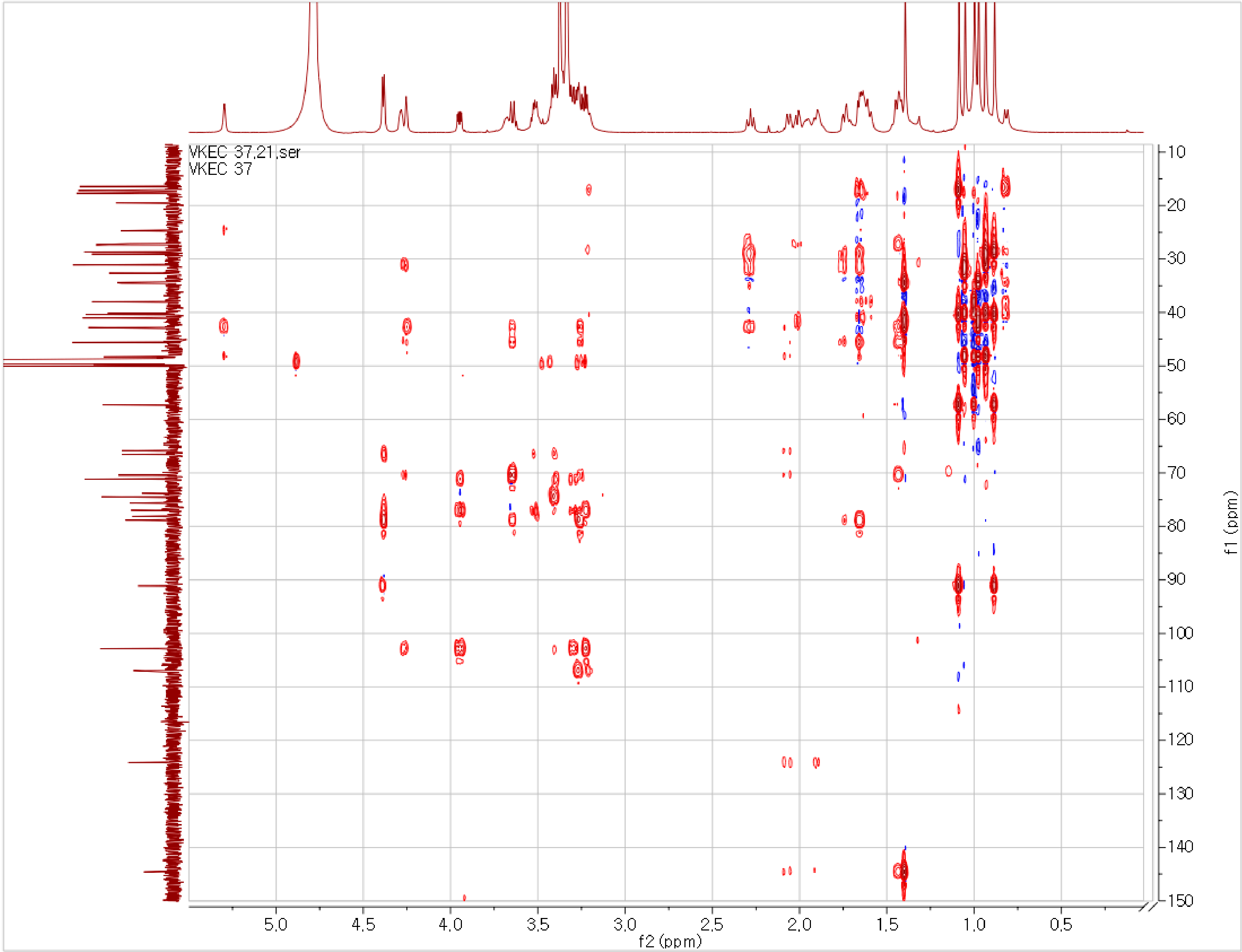

Figure S7. The NOESY spectrum of **1** in methanol-d_4

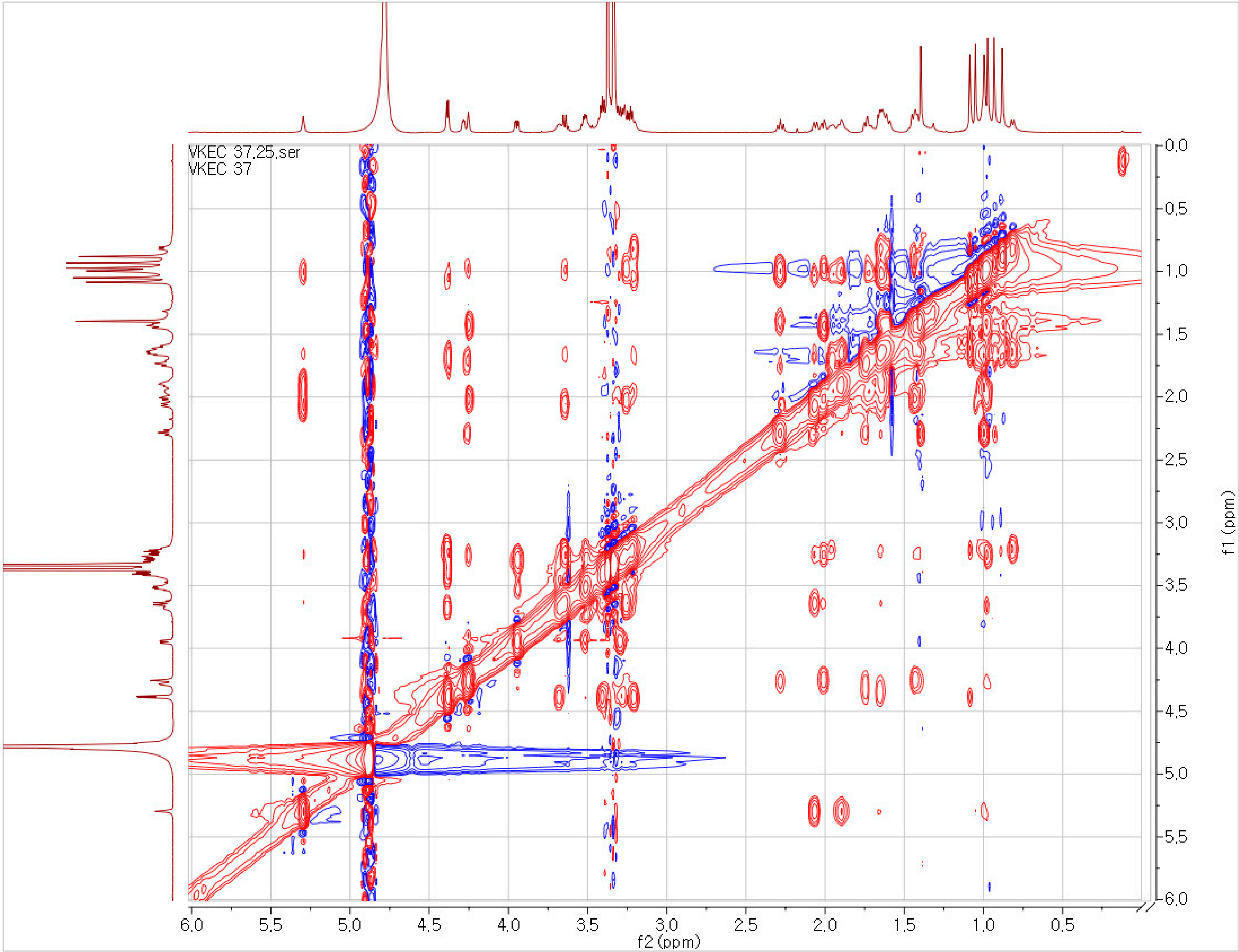

Figure S8. The DEPT90 (top) and 135 (bottom) spectra of **1** in methanol-d_4

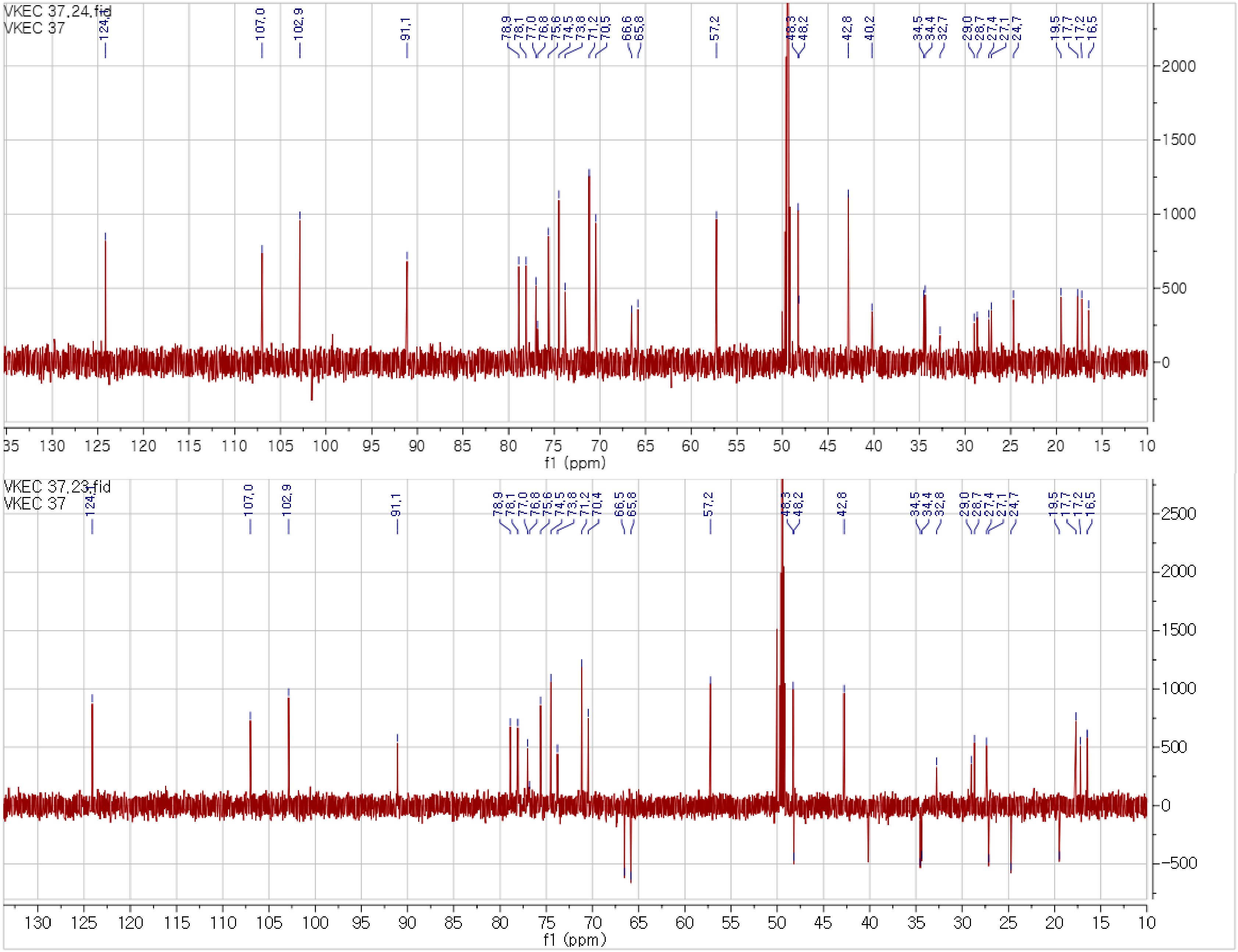

Figure S9. The HRFABMS spectrum of **2**

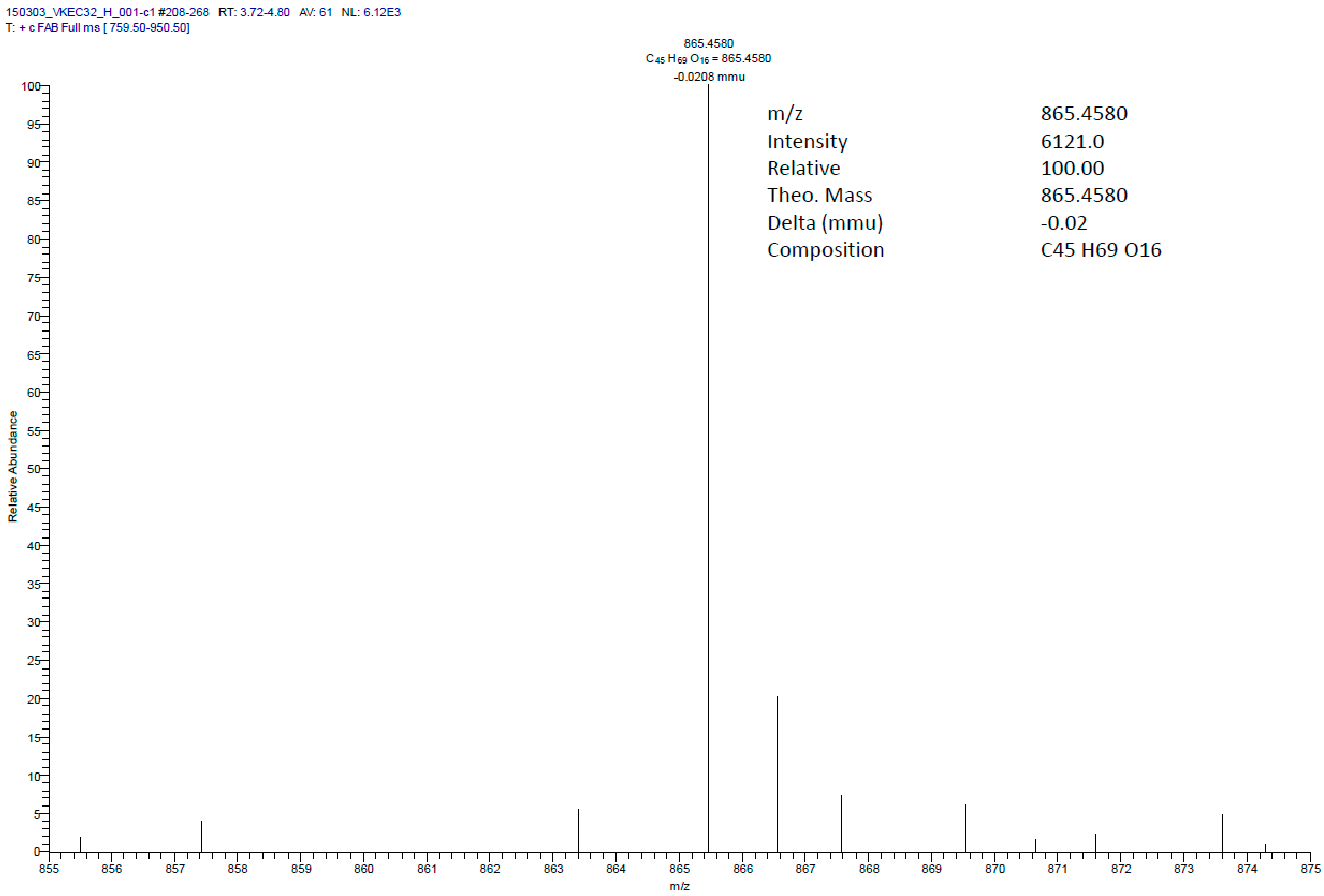

Figure S10. The ^{1}H NMR spectrum of **2** in methanol-d_4

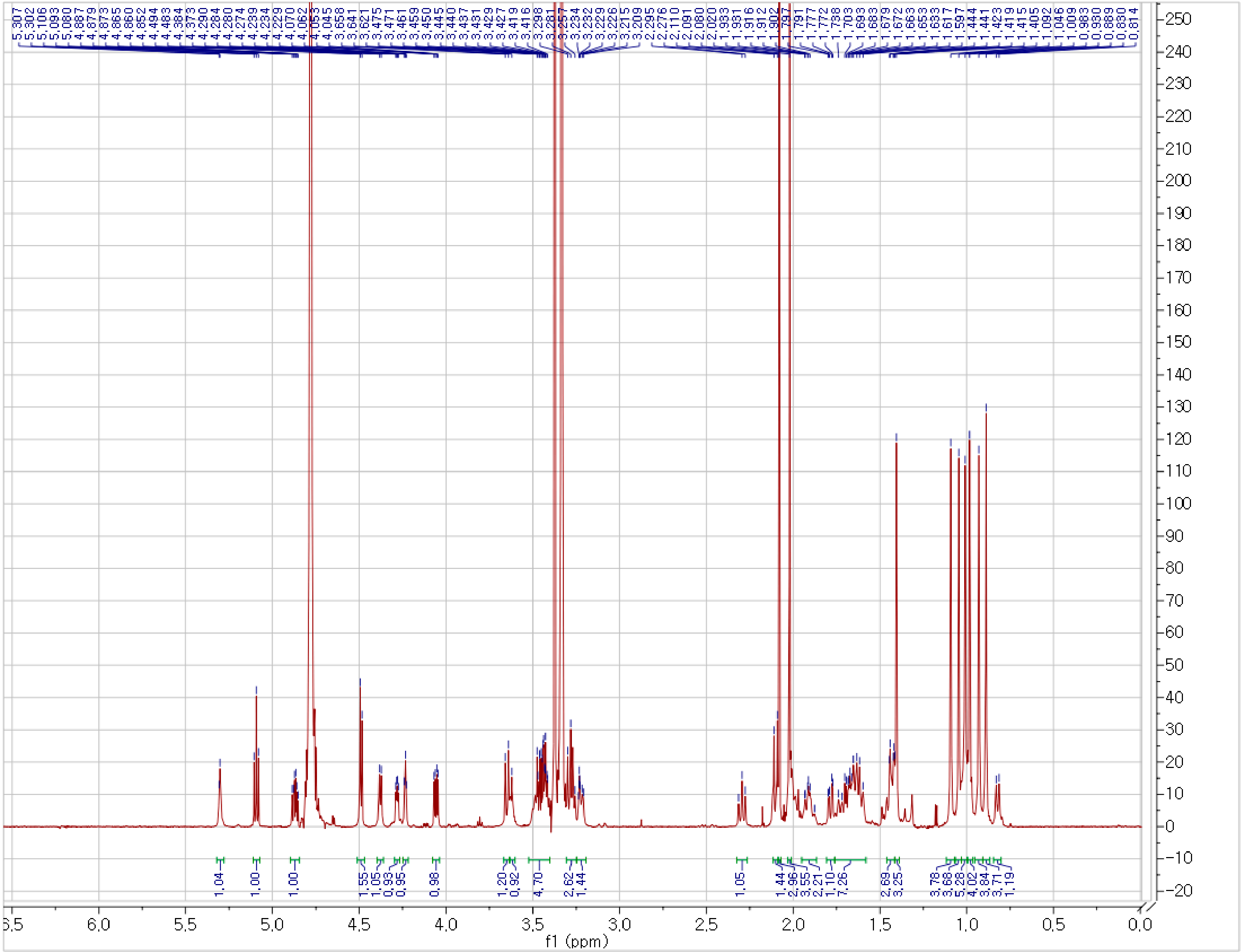

Figure S11. The ^{13}C NMR spectrum of **2** in methanol-*d*$_4$

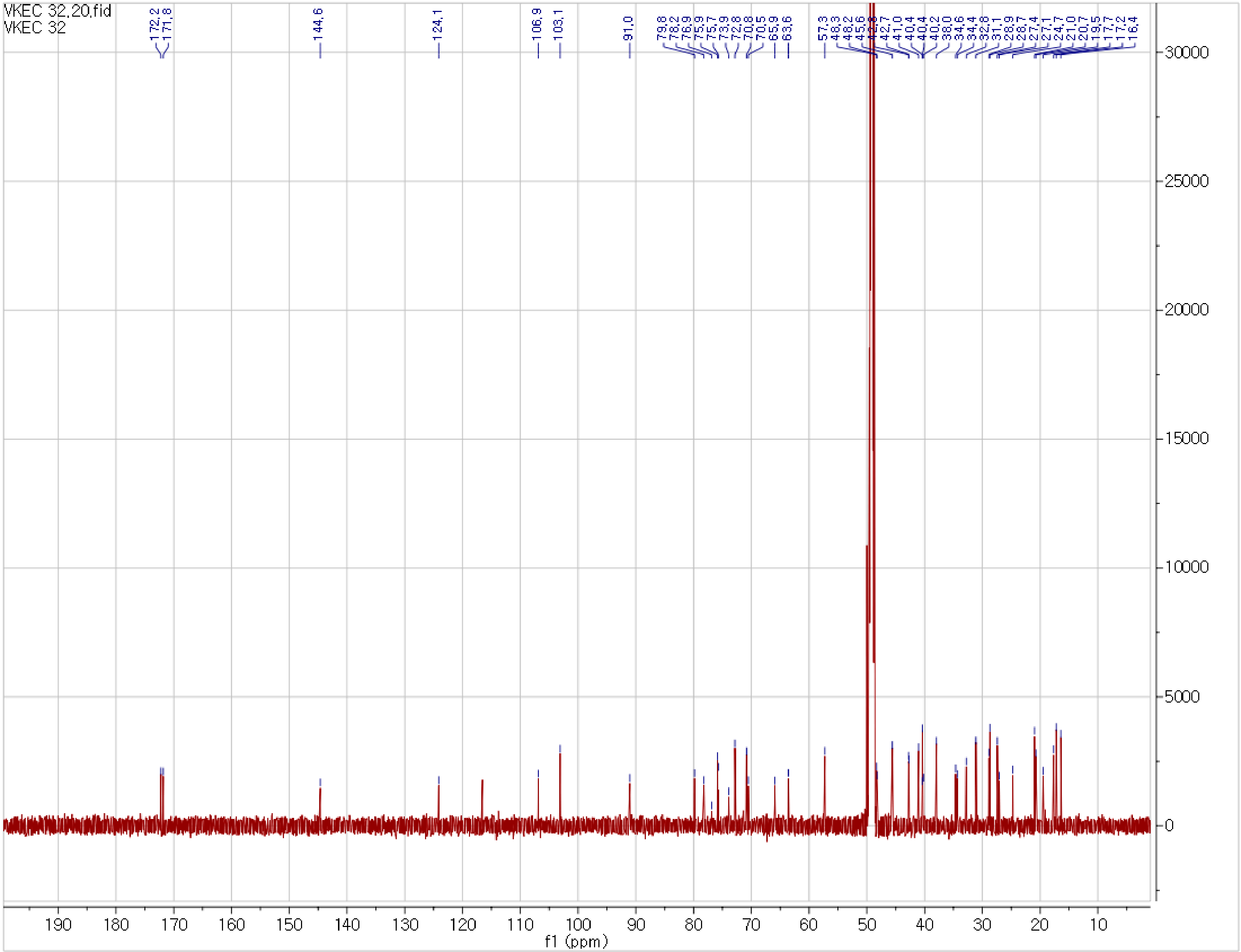

Figure S12. The COSY spectrum of **2** in methanol-d_4

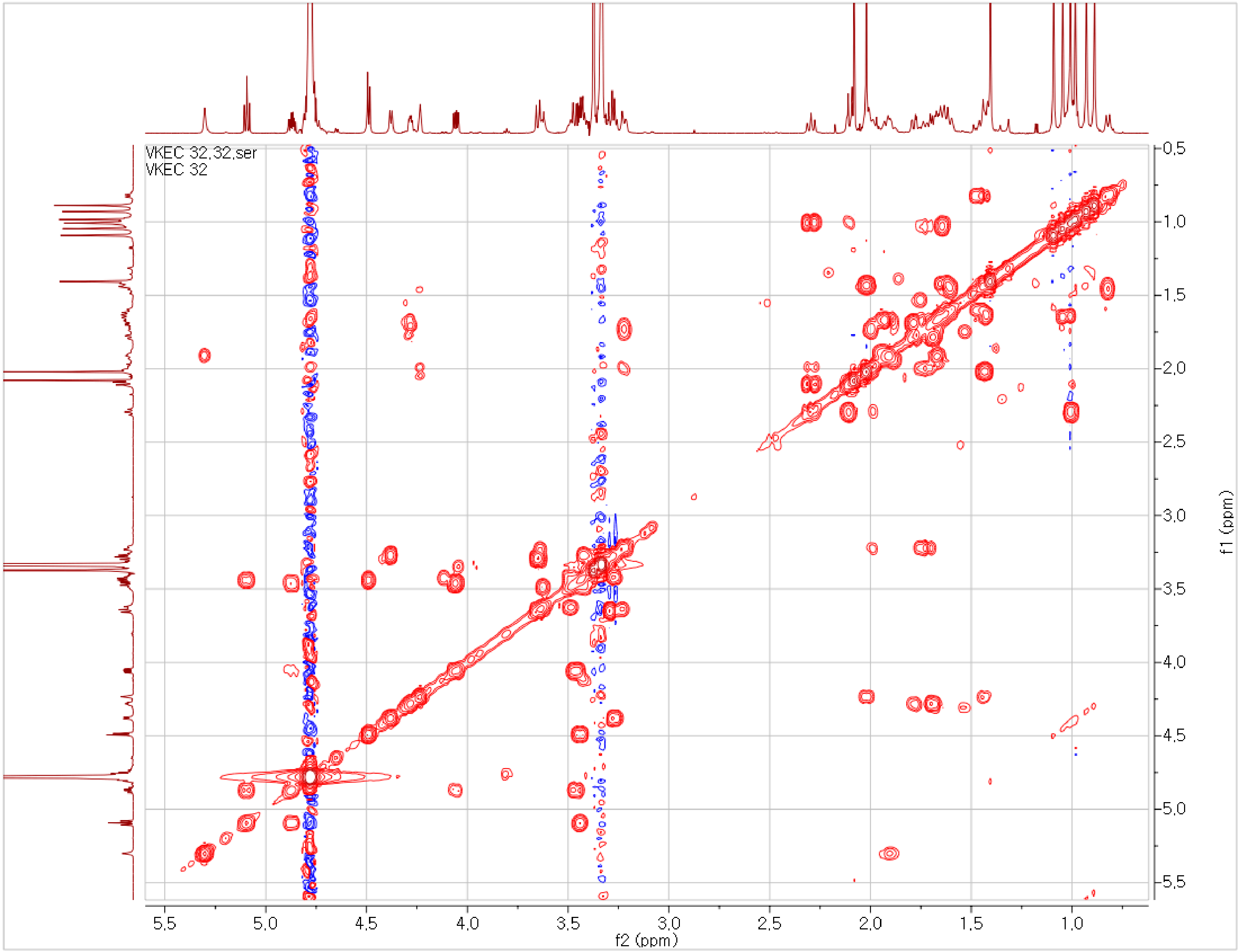

Figure S13. The HSQC spectrum of **2** in methanol-d_4

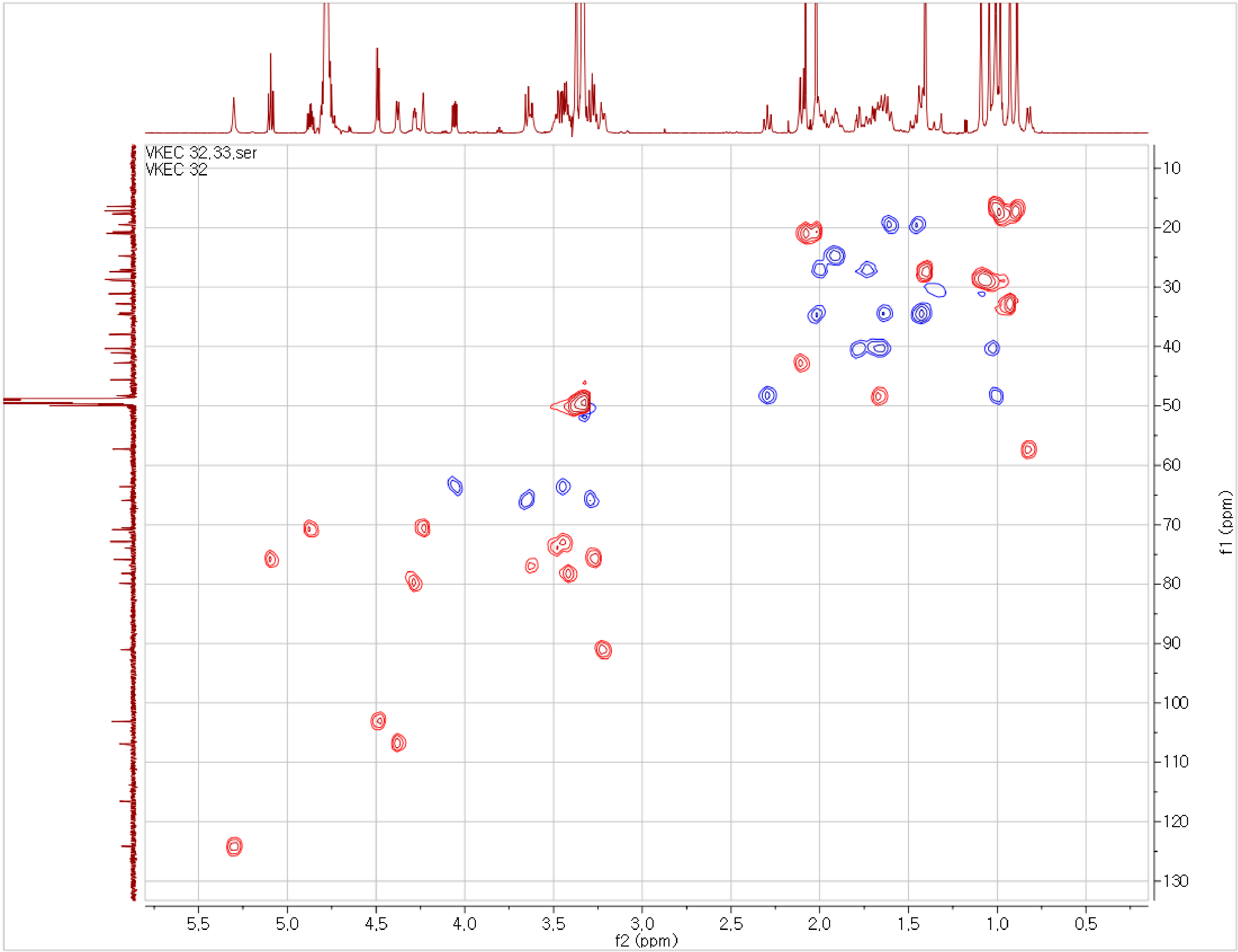

Figure S14. The HMBC spectrum of **2** in methanol-d_4

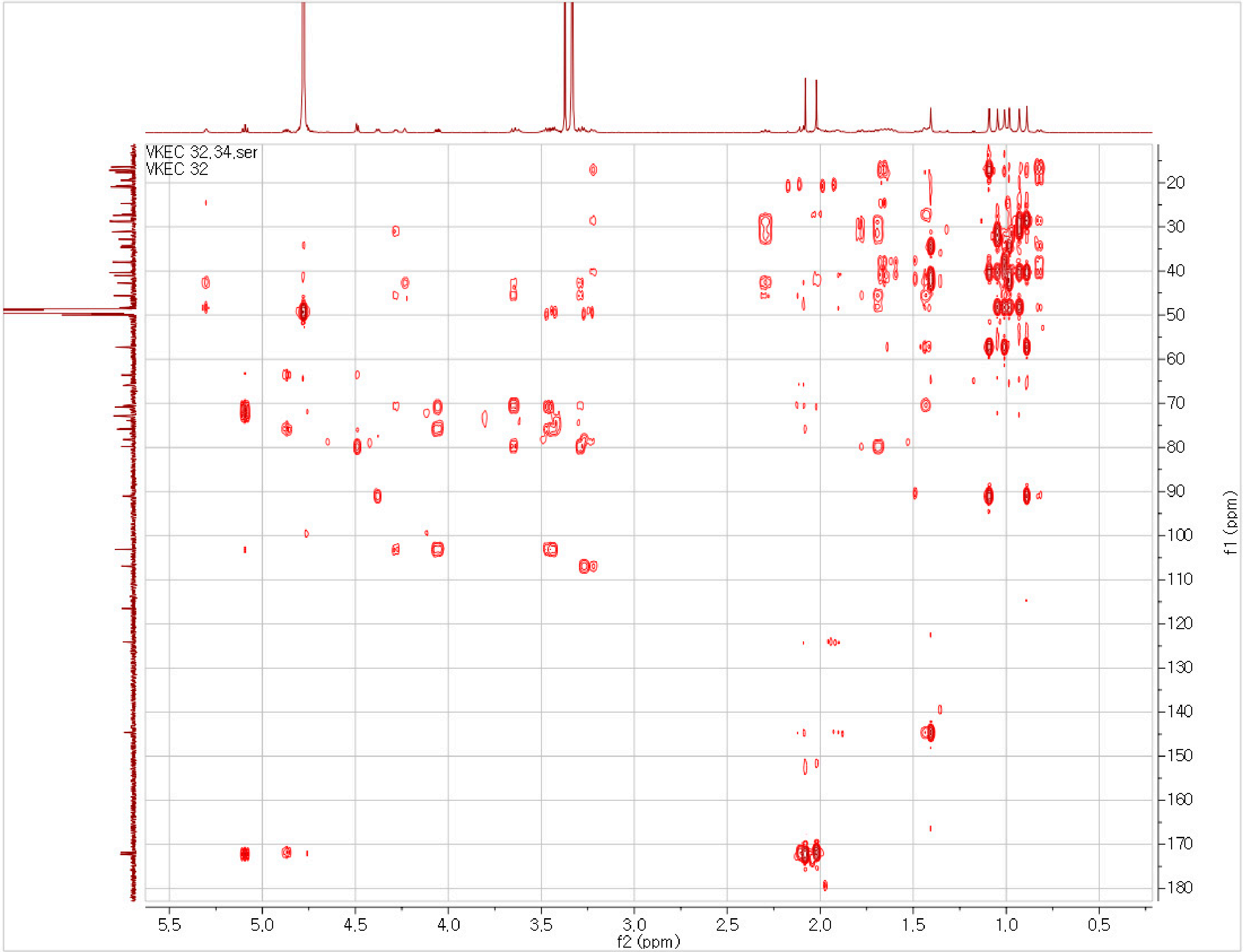

Figure S15. The NOESY spectrum of **2** in methanol-*d*4

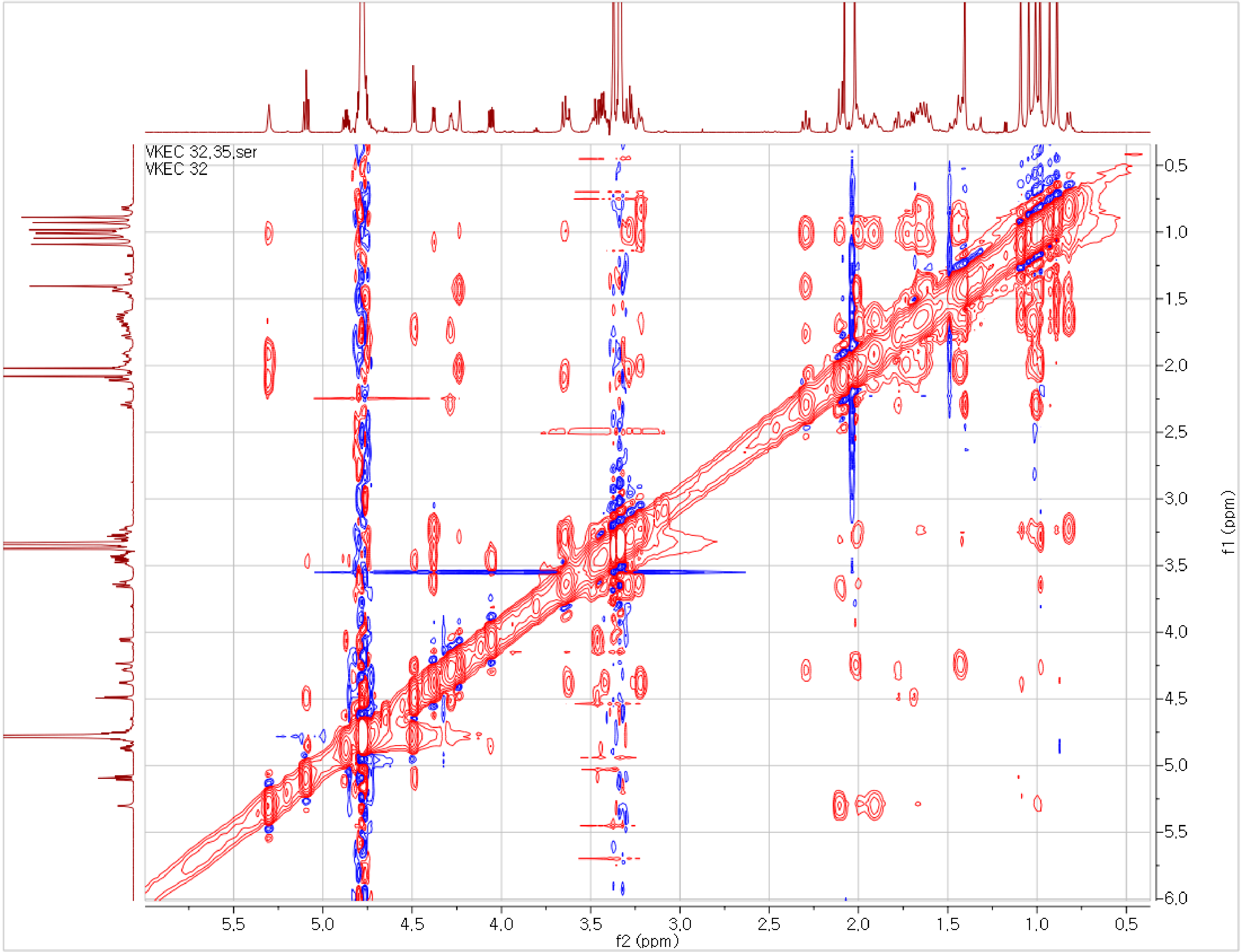

Figure S16. The HRFABMS spectrum of **3**

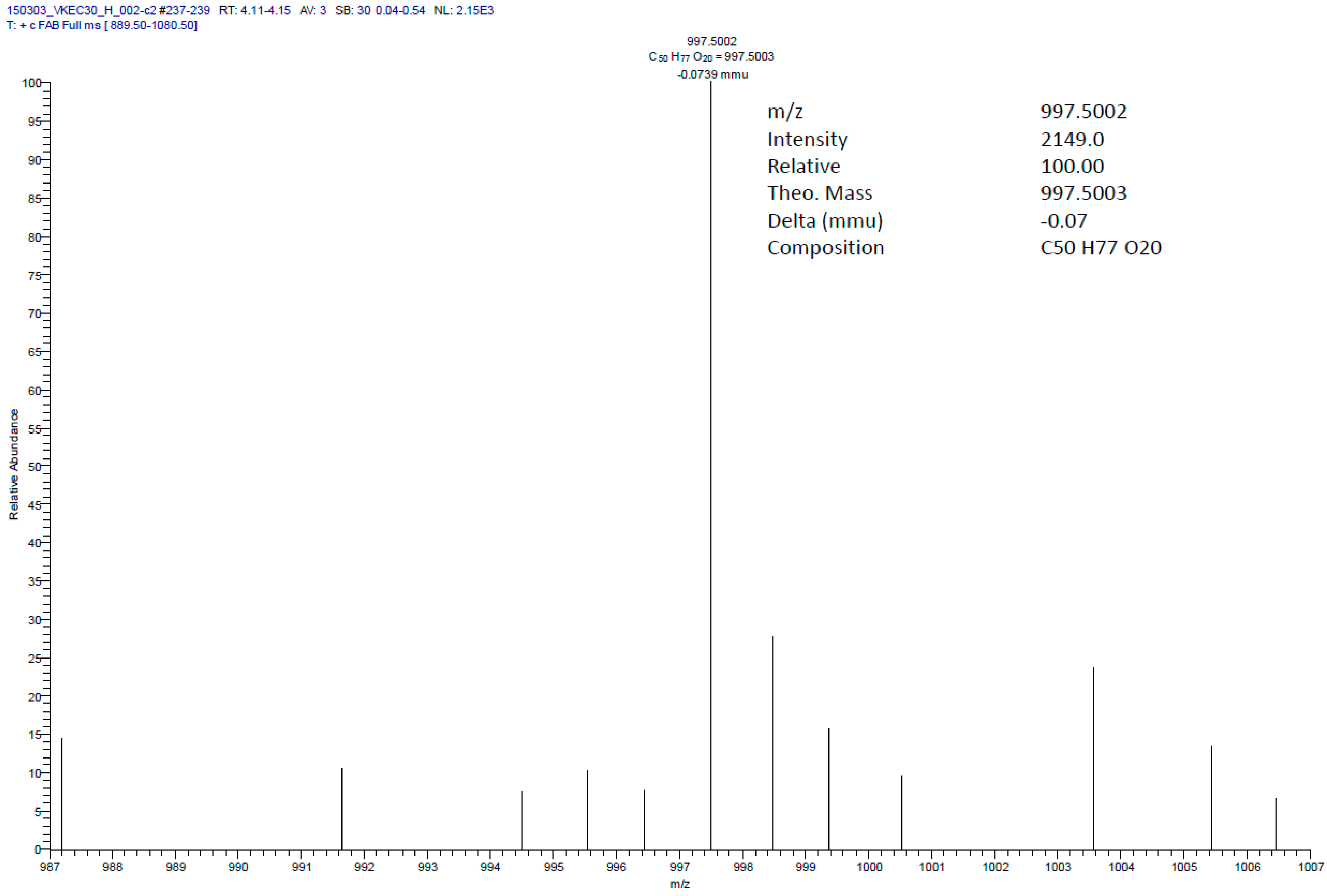

Figure S17. The ^{1}H NMR spectrum of **3** in methanol-d_4

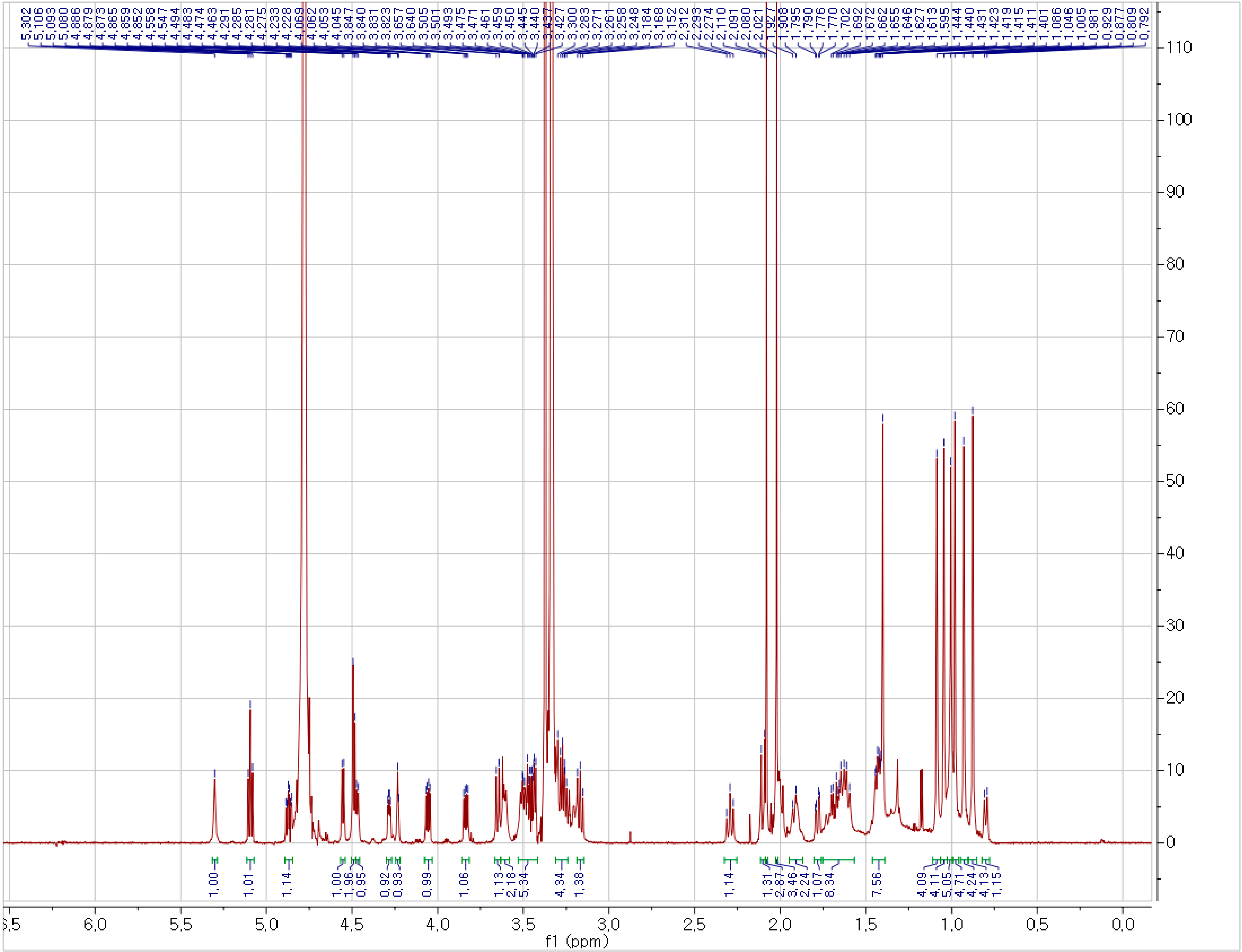

Figure S18. The ^{13}C NMR spectrum of **3** in methanol-*d*4

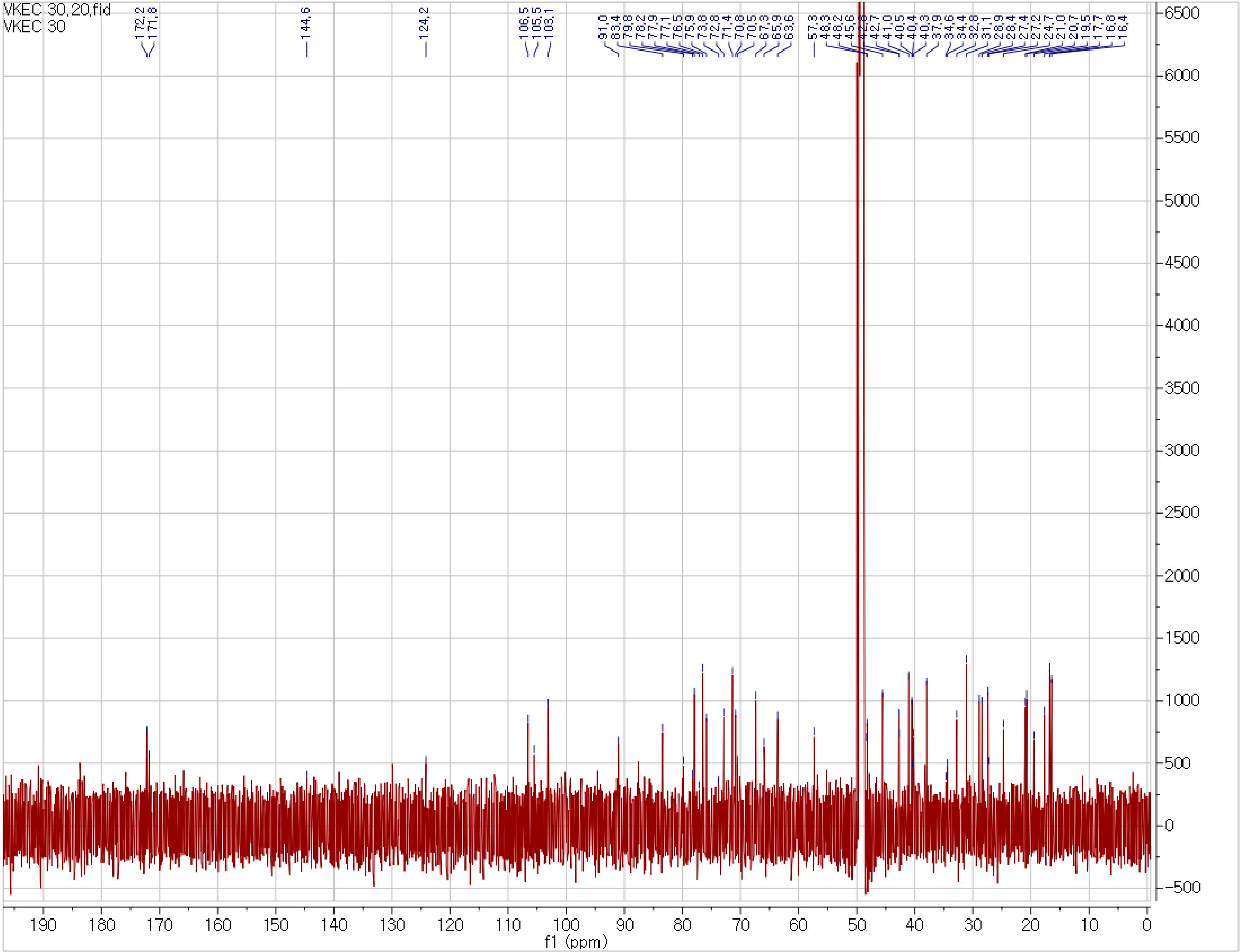

Figure S19. The COSY spectrum of **3** in methanol-d_4

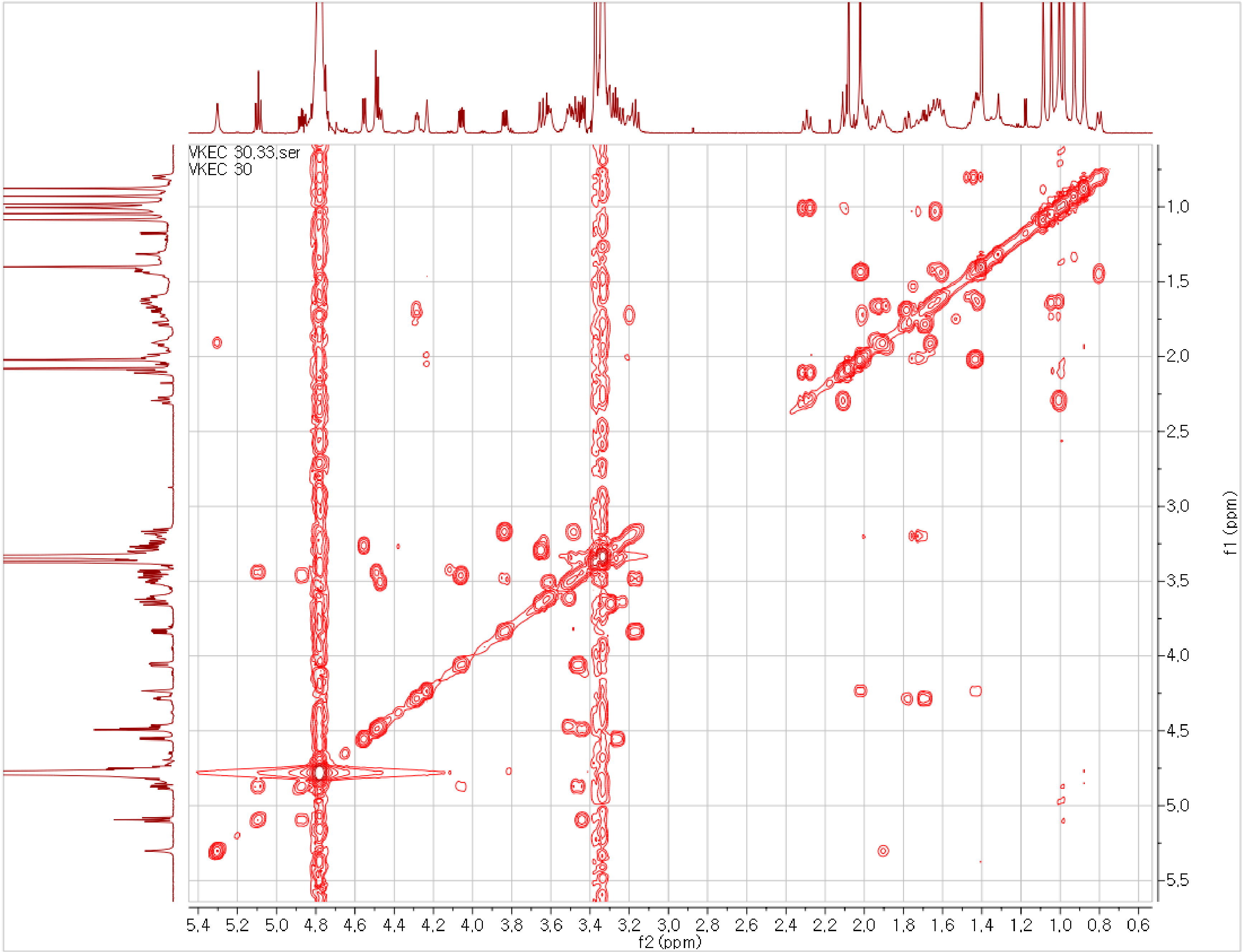

Figure S20. The HSQC spectrum of **3** in methanol-d_4

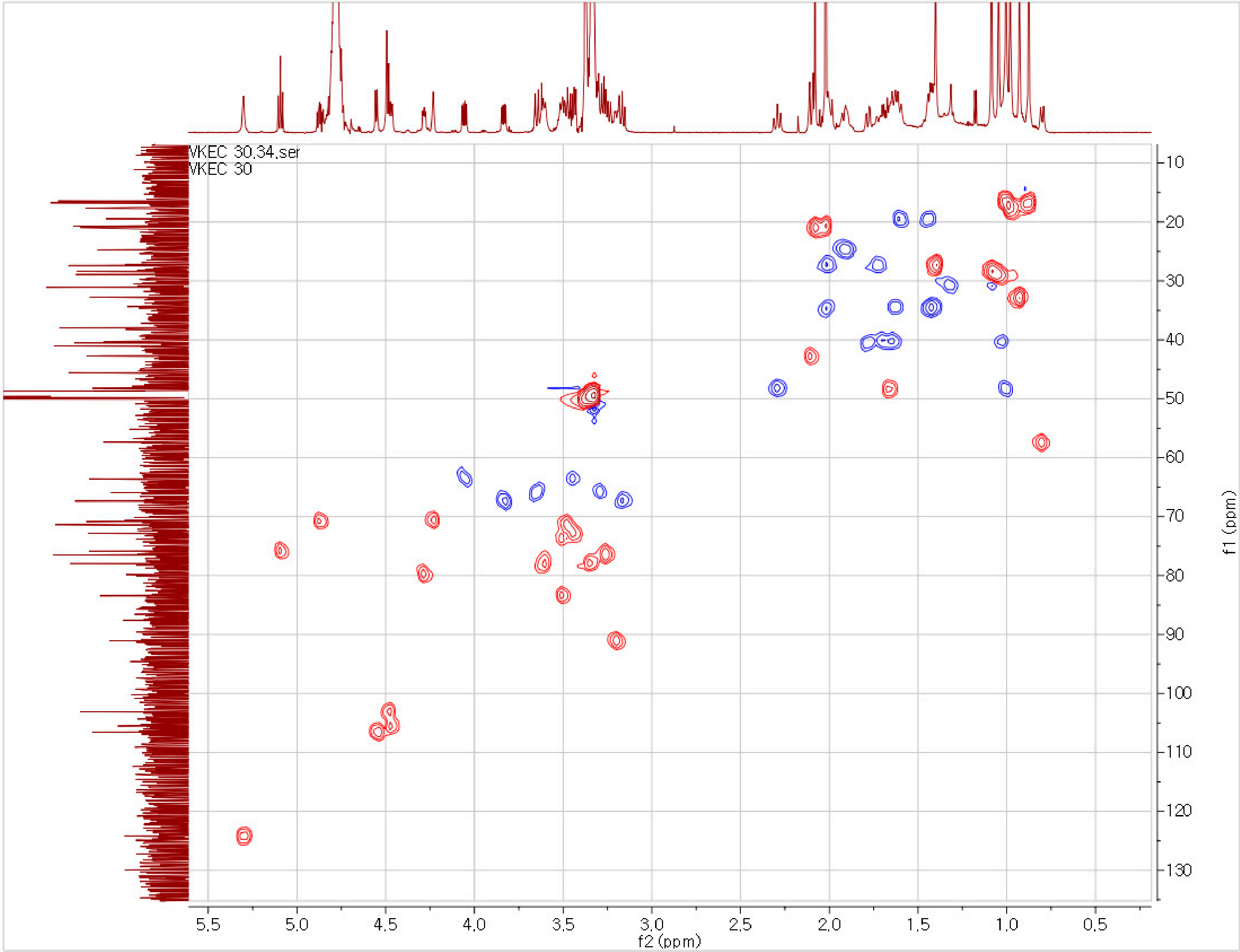

Figure S21. The HMBC spectrum of **3** in methanol-d_4

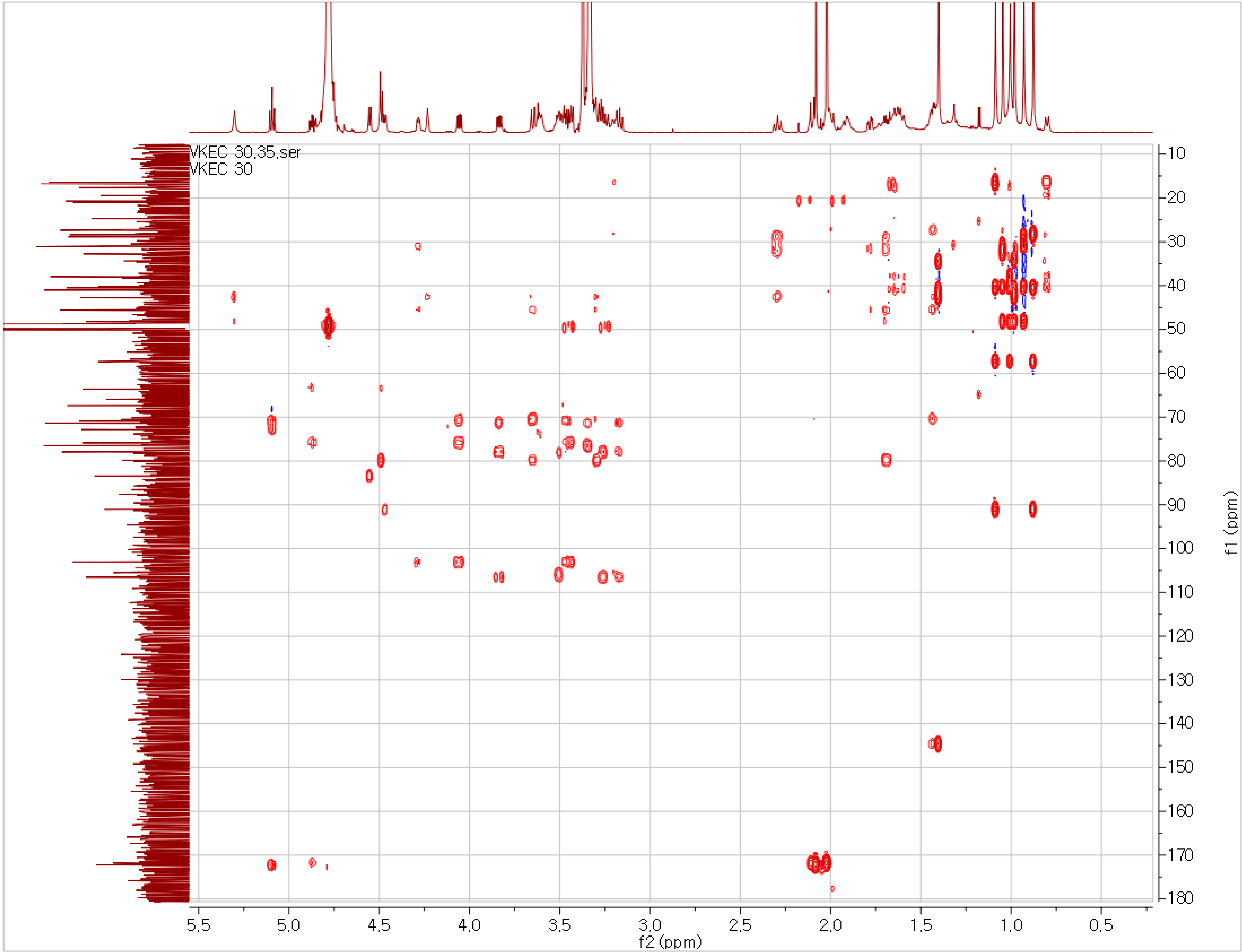

Figure S22. The NOESY spectrum of **3** in methanol-d_4

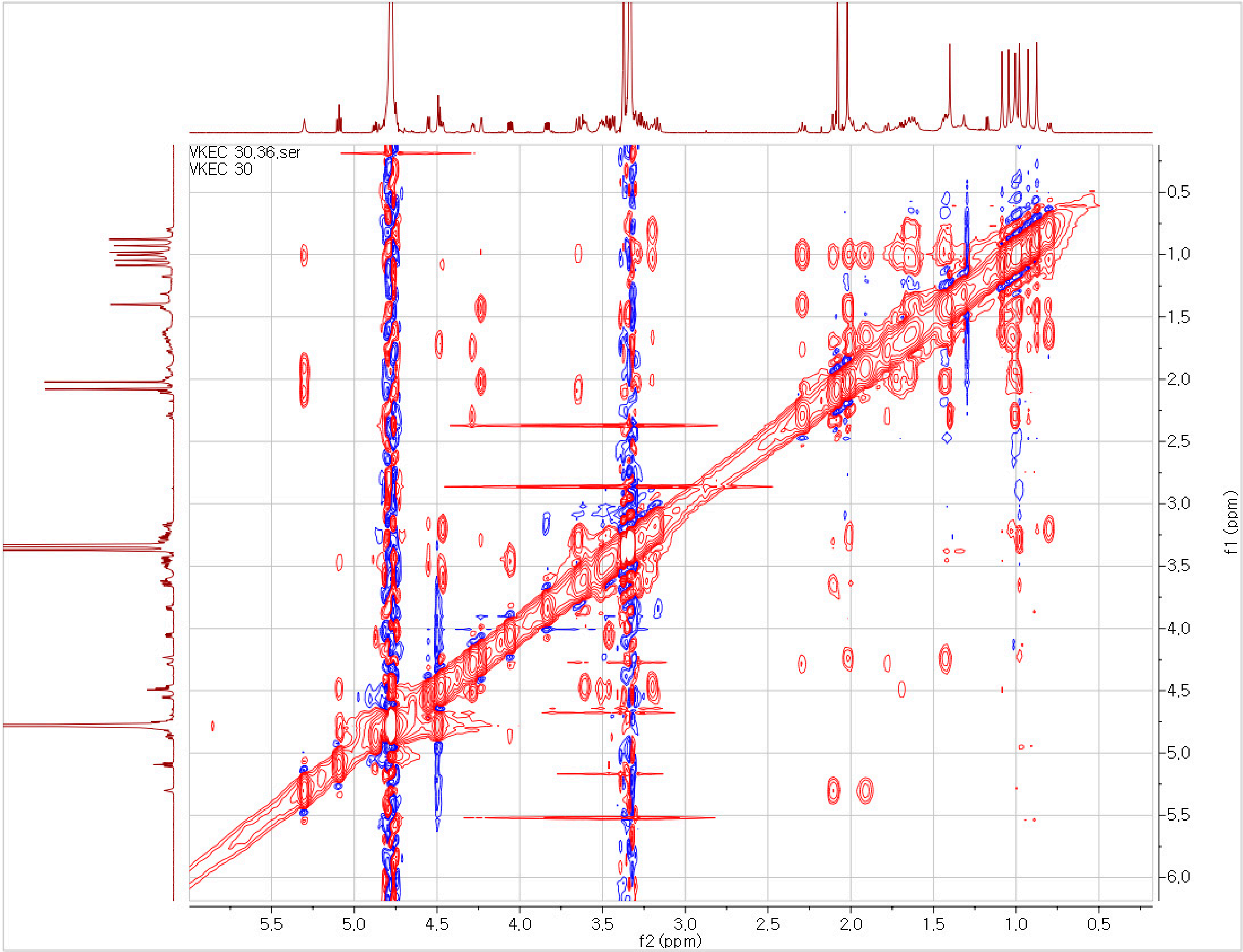

Supplement: Supplementary file 1 [file plants-09-01083-s001.pdf]
